# Thermo-Convective Solution Growth of Vertically Aligned Zinc Oxide Nanowire Arrays for Piezoelectric Energy Harvesting

**DOI:** 10.3390/mi15101179

**Published:** 2024-09-24

**Authors:** Frank Eric Boye Anang, Andam Deatama Refino, Gunilla Harm, Defang Li, Jiushuai Xu, Markys Cain, Uwe Brand, Zhi Li, Marion Görke, Georg Garnweitner, Erwin Peiner

**Affiliations:** 1 Institute of Semiconductor Technology, Technische Universität Braunschweig, 38106 Braunschweig, Germany; a.refino@tu-braunschweig.de (A.D.R.); g.harm@tu-braunschweig.de (G.H.); defang.li@tu-braunschweig.de (D.L.); jiushuai.xu@tu-braunschweig.de (J.X.); e.peiner@tu-braunschweig.de (E.P.); 2 Scientific Metrology Department, Ghana Standards Authority (GSA), Accra P.O. Box MB 245, Ghana; 3 Electrosciences Ltd., Farnham GU9 9QT, Surrey, UK; markys.cain@electrosciences.co.uk; 4 Surface Metrology Department, Physikalisch-Technische Bundesanstalt (PTB), 38116 Braunschweig, Germany; uwe.brand@ptb.de (U.B.); zhi.li@ptb.de (Z.L.); 5 Institute for Particle Technology, Technische Universität Braunschweig, 38104 Braunschweig, Germany; m.goerke@tu-braunschweig.de (M.G.); g.garnweitner@tu-braunschweig.de (G.G.); 6 Laboratory for Emerging Nanometrology (LENA), Technische Universität Braunschweig, 38106 Braunschweig, Germany

**Keywords:** thermo-convective solution growth, ZnO nanowire arrays, seed layer, piezoelectric energy harvesting, XRD, Raman spectroscopy

## Abstract

The search for a synthesis method to create longer ZnO NWAs with high-quality vertical alignment, and the investigation of their electrical properties, have become increasingly important. In this study, a hydrothermal method for growing vertically aligned arrays of ZnO nanowires (NWs) using localized heating was utilized. To produce longer NWs, the temperature environment of the growth system was optimized with a novel reaction container that provided improved thermal insulation. At a process temperature above ~90 °C, ZnO NWs reached a length of ~26.8 µm within 24 h, corresponding to a growth rate of 1.1 µm/h, nearly double the rate of 0.6 µm/h observed in traditional chemical bath growth using a glass reactor. The densely grown NWs (~1.9/µm^2^), with a diameter of ~0.65 µm, exhibited a preferred hexagonal *c*-axis orientation and were vertically aligned to the (100) silicon (Si) substrate. These NW structures have multiple applications, e.g., in piezotronic strain sensors, gas sensing, and piezoelectric energy harvesting. As proof of concept, a piezoelectric nanogenerator (PENG) was fabricated by embedding the NWs in an S1818 polymer matrix over a 15 mm × 15 mm area. Under repeated impulse-type compressive forces of 0.9 N, a maximum peak output voltage of ~95.9 mV was recorded, which is higher by a factor of four to five than the peak output voltage of 21.6 mV previously obtained with NWs measuring ~1.8 µm in length.

## 1. Introduction

ZnO is a common *n*-type semiconductor material that has an extended 3.37 eV energy band gap and high exciton binding energy of 60 meV [1,2]. It is a material that is easily processed, widely accessible, and non-toxic. ZnO nanowires (NWs) have been studied in numerous applications, including piezotronic strain sensors [3], dye-sensitized solar cells [4,5], UV photodetectors [6], gas detection devices [7,8], and piezotronic transistor arrays [9]. ZnO has also been proven to be a perfect material for creating energy-conversion devices, due to its unique ability to couple its semiconducting and piezoelectric properties, thereby resulting in an exceptionally efficient transformation of mechanical energy into electrical energy [10,11,12]. Moreover, because of their affordability, wide usage, great biocompatibility, and favorable optoelectronic properties, ZnO piezoelectric materials are largely preferred among other one-dimensional (1D) semiconductor materials [5,13,14].

Energy harvesting in micro- and nanoscale applications for small and wireless autonomous devices, also known as nanogenerators (NGs), is based on a technology that transforms mechanical/thermal energy, produced by minute physical changes, into electrical energy. The technology can be divided into three main categories, namely, triboelectric nanogenerators (TENGs), pyroelectric nanogenerators (PyENGs), and piezoelectric nanogenerators (PENGs) [15].

The operation of TENGs is based on the coupling effect of electrostatic induction and frictional electrification, which leads to an electron transfer between two materials and the collection of the resultant electrical energy generated [16,17,18]. Two types of friction modes are involved, which are the sliding mode and contact separation mode. While they provide the benefits of safety, low cost, light weight, and environmental friendliness, their susceptibility to environmental changes limits the extent of their application [15,18].

To transform external thermal energy into electrical energy, PyENG devices use nanostructured pyroelectric materials. There are two distinct scenarios in which the working principle of pyroelectric nanogenerators can be explained. The first is based on temperature fluctuations in pyroelectric materials that are constantly under mechanical strain to prevent expansion or contraction. The second correlates to the thermal strain of applied temperature changes and is caused by the pyroelectric material’s deformation [19,20]. PyENGs are extremely vulnerable to temperature changes in the environment, despite the fact that they also offer extensive application potential in self-powered and autonomous systems [20,21].

The exchange of mechanical and electrical energy in dielectric materials is known as the piezoelectric effect in PENGs. There are two types of piezoelectric effects: the inverse piezoelectric effect, which transforms electrical energy into mechanical energy, and the direct piezoelectric effect, which converts mechanical energy into electrical energy. The utilized material has the effect of linking the electric field and the stress field because of the unique arrangement of atoms in the piezoelectric crystal lattice. The output voltage and current of piezoelectric ZnO NWs can be enhanced by the integration of several NGs in series or parallel, respectively [22]. In a recent study, we proposed a patterned growth of ZnO NWs that can be integrated in series and in parallel to enhance the piezoelectric voltage and current, respectively [23]. To further improve the overall output of the PENG, it is beneficial to use vertically aligned and ultra-long NWs [24,25], which is the primary focus of this work. Generally, PENGs have a long service life, are less impacted by environmental conditions during operation [26], and produce stable output under repeated compressive force applications [14].

Despite these advantages, there is still room for improvement. Differing ZnO NW shapes and array densities can significantly affect piezoelectric output. As simulations and experiments have demonstrated, piezoelectric output will be improved by higher aspect ratio and density [27,28,29]. Furthermore, parameters such as growth temperature [30], growth time [31], and seed layer thickness [32] have significant influence on ZnO NWA morphology. Consequently, an important area of research is the realization of ZnO NW arrays (NWAs) with morphologies that can increase piezoelectric output. ZnO NWAs can currently be produced using a variety of techniques, such as chemical bath deposition (CBD), also known as the hydrothermal method [6,11,33], metal–organic chemical vapor deposition (MOCVD) [8], and thermal evaporation [1]. While methods other than hydrothermal synthesis are effective, they are capital intensive and require higher processing temperatures (400–900 °C), which are not conducive to many flexible device applications [34]. Hydrothermal synthesis, on the other hand, is one of the most effective ways to minimize costs [35] because it does not require harsh conditions like high temperatures and pressures or environments resistant to corrosion. It can generally be carried out under normal atmospheric pressure, at temperatures below 100 °C, and can also be performed on different substrates for various applications.

Although ZnO NWAs can be grown on a variety of substrates, including carbon fibers, glasses, Si wafers, metals, and flexible printed circuit boards (PCBs) [5,36], the NW morphology can still vary slightly under the same conditions [36]. Si substrates have become the standard option due to their strong interoperability with other circuit systems in subsequent applications. ZnO NWAs produced on Si substrates without a ZnO seed layer (SL) tend to develop in an extremely haphazard manner and typically do not generate a homogeneous film. On the other hand, by using a substrate with a ZnO SL, NWs can be controlled to grow perpendicular to the substrate, or along the ZnO crystal’s *c*-axis. In this study, we used sputtered elemental Zn, which is then annealed in a normal air ambience for oxidation to form ZnO. Both the density and the length uniformity of the NWAs are influenced by this annealing process and the resulting SL thickness. This has been demonstrated well in the work of Jiushuai Xu et al. [37]. With an annealing temperature set at 600 °C and an annealing period of 1 h, the NWAs had the optimal *c*-axis orientation, perpendicular alignment, and the largest density. Other groups have reported the direct deposition of ZnO SLs via sputtering [38,39], spin-coating [40], and dip coating [3]. However, for patterned growth of ZnO NWAs, necessary for easy integration into microelectromechanical systems (MEMS) fabrication processes (e.g., piezoresistive cantilever spring-mass resonators [41]), these methods are less favorable. Therefore, in this study, we introduce a two-step ZnO NWA growth method using DC sputtering of Zn metal, followed by an annealing process in air ambience at a temperature of 600 °C, to form a ZnO seed layer (SL). By employing this Zn sputtering technique, devices requiring patterning by lift-off of the Zn precursor layer using photoresist for area-selective growth of ZnO NWAs [23] can easily be processed. Our study focuses on investigating the impact of ZnO SL thickness on the morphology of ZnO NWs and the subsequent fabrication of ZnO-based PENGs using ultra-long NWs realized in this study.

## 2. Materials and Methods

### 2.1. Investigation of SL Thickness on Morphology of ZnO NWAs

The ZnO SL serves as a foundation for NW growth and is crucial to the arrangement of NWAs. The ZnO NWs’ diameter, growth direction, and regularity of vertical arrangement are all determined by the thickness of the SL [2,42]. The mass transport of chemical precursors limits the density of NWs per unit area [42]. The length of the NWs gradually drops, and the diameter increases as the SL increases in thickness [42,43]. The arrangement of aligned NWs in arrays is improved when the thickness of the SL increases, the size of the crystal grains grows, and the frequency of crystal-grain roughness of the SL on the substrate surface decreases. This causes the NWs to be more inclined to grow vertically [44]. Furthermore, the configuration of the NWs will be impacted by the annealing process used for the SL. ZnO NWs are orientated arbitrarily because of a substantial lattice mismatch caused by crystal rearrangement and recrystallization between the NWAs and the thick, unannealed ZnO SL. The disassociated ZnO atoms are given enough energy during the annealing process to form a more stable structure, which increases the number of nucleation sites and improves crystallinity, as well as the electrical characteristics of the material. The annealing process enhances the ZnO SL’s adherence and crystallinity on the substrate, which helps to generate well-aligned ZnO NWAs [45]. In this experiment, we investigated the influences of SL thickness on the morphology (NW length, diameter, aspect ratio, density, and alignment) of long ZnO NWs.

The deposition of ZnO SLs was preceded by preparing an *n*-type P-doped Si substrate with <100> crystal orientation and measuring 15 mm × 15 mm, which was employed in the experiment. It has a thickness of 525 ± 20 µm and 1–5 Ω·cm resistivity. To remove any metal and organic pollutants that may be present on the Si surface, the material was first submerged in a solution containing 96% sulfuric acid (H_2_SO_4_) and 30% hydrogen peroxide (H_2_O_2_) at 110 °C in a volume ratio of 1:1 for 5 min. The natural oxide layer of SiO_2_ that had grown on the surface was then cleaned for 10 min using hydrofluoric acid (HF) diluted to 6–7% in H_2_O by volume. The sample was then rinsed in DI water and dried with a stream of nitrogen (N_2_) gas. The ZnO SL deposition was carried out by conducting DC sputtering of a Zn metal target with 99.99% purity [23]. The sputtering was conducted using an S150B sputter coater (supplied by HHV Ltd., Crawley, UK) at varying sputtering times (1 to 40 min). This was followed by annealing in air at 600 °C for 1 h to complete the formation of ZnO. Prior to DC sputtering of Zn metal, a display foil was used to mask one half of the Si substrate to create a step for subsequent ZnO thickness measurement. Following the DC sputtering, the display foil was removed and the Si sample annealed. The detailed ZnO SL deposition process has been reported in our earlier publications [23,37].

### 2.2. Temperature Optimization for Experimental Setup

Studies have demonstrated that enhanced thermal convection and local heating can be used to grow superior vertically oriented ZnO NWAs. In our first attempts to realize a thermo-convective solution growth (TSG) setup, we were able to generate ZnO NWs with an overall length of ~30 µm only after 72 h, exhibiting a vertical growth rate of 0.42 µm/h, i.e., much lower than 2 μm/h as expected [3]. Variations in the processing of the experimental setup itself can produce disparate outcomes. The built-in heating system in our previous setup has been identified to have faced two important challenges:

1. The volume of the reaction container was large, resulting in a large surface area in contact with the cooling water. This was detrimental to the temperature stability required for the precursor solution. Also, the external cooling system’s effectiveness (together with the cooling water) was higher than the heating element’s heating efficiency. As a result, it was challenging to control and maintain a stable growth temperature.
2. The temperature achieved in the reaction container differed from the input temperature shown on the temperature controller. This was because the PT100 temperature sensor that was utilized in the heating system was mounted inside the heating element rather than on its surface. Consequently, there were additional materials with increased thermal resistance between the thermal resistor and the PT100. These included pore space at the interface between the heating element and the solution, a 2 mm thick aluminum sheet for hermetic packaging, graphite sheets to aid heat dissipation, and a specified thickness of the Si substrate.

The challenges mentioned above have necessitated the optimization of the current setup to realize longer and vertically aligned ZnO NWAs. This article therefore proposes that two techniques be considered. One is to use a thermocouple (TC) sensor in contact with the heating element so that the actual temperature between the heating element and the substrate (Si) can be determined. The other is to investigate the thermal conductivity in different reaction containers vis-a-vis the temperature of the precursor solution. These details are described in the Supplementary Materials (see Supplementary Notes S2–S4).

### 2.3. ZnO NWA Growth

Based on the findings from the above experimental investigations, ZnO NWAs were fabricated using the newly developed growth setup. Zinc nitrate hexahydrate (Zn(NO_3_)_2_) from Alfa Aesar corporation (molar mass: 297.47 g/mol, purity: 99%) and hexamethylenetetramine (HMT) from Sigma Aldrich corporation (Burlington, MA, USA) (molar mass: 140.19 g/mol, purity: ≥99.0%) were the reagents used for the ZnO NW growth experiment. Without needing to be further purified, both reagents were ready for use. To ensure that the two reagents were thoroughly mixed, they were dissolved in 250 mL of DI water, each at a concentration of 0.025 M (1:1), and agitated for 90 s with a glass rod. The HMT primarily functions as a pH buffer by slowly releasing OH^−^ ions through thermal degradation, which aids in the formation of ZnO nanostructures during the hydrothermal process [46]. Details of the hydrothermal growth process can be found in the Supplementary Materials (see Supplementary Note S1).

**Figure 1:**
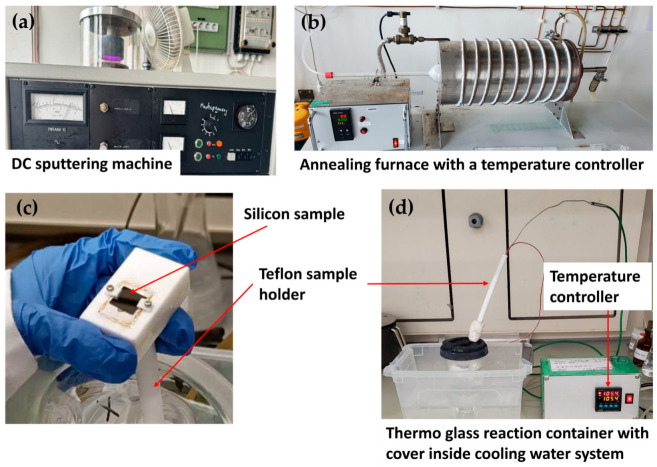
Experimental setup for ZnO NWA growth by thermo-convective solution growth method: (**a**) machine for DC sputtering of a Zn target onto a Si substrate; (**b**) annealing furnace for oxidizing the Zn metal layer sputtered onto Si substrate at 600 °C for 1 h to form a ZnO seed layer (SL); (**c**) Si sample with ZnO SL affixed to a Teflon sample holder; (**d**) Sample immersed in a thermo glass reaction container filled with precursor solution for ZnO NWA growth by CBD or TSG.

To guarantee that the sample would not slide off during the reaction, the cleaned Si substrate was fixed on the surface of the heating element using tiny splints on both sides of the element (Supplementary Figure S1b). The Teflon holder with the heating element was then submerged in the container at a 45° angle. This ensured that a path was created for the gas generated during the reaction, to allow the air bubbles formed in the process to flow out. Subsequently, the reaction container was covered and submerged in the cooling water to stop global heating and the nutrient intake that results from running the experiment over longer hours. The ZnO NW growth was performed at *T*_input_ (95 °C, 100 °C, and 105 °C) (see Supplementary Figure S2), for a growth period of 24 h. Following the growth of the ZnO NWs, the samples were cleaned with acetone in an ultrasonic bath for 3 min, followed by isopropanol, to rid them of any contaminants that may have adhered to the substrate surface. Lastly, the samples were dried with a stream of N_2_ gas. Figure 1 depicts the experimental setup for the ZnO NWA fabrication process in this study.

### 2.4. Characterization of ZnO Crystal Structures

The crystal structure and orientation of the ZnO SL and ZnO NWAs were measured using X-ray diffraction (XRD). XRD is commonly applied to characterize crystalline materials and provide information about the crystalline structure, nature of present phases, lattice parameters, strains, and crystalline grain size. The XRD measurements in this study were performed using an Empyrean series 2 diffractometer (MalvernPanalytical, Almelo, The Netherlands) with Cu Kα radiation (*λ* = 0.15419 nm, *Kα*_2_: *Kα*_1_ = 0.5, Cu LFF HR as X-ray source) and a PIXcel3D detector. Diffractograms were taken in the range of 2*θ* = 20–90° with a step size of 0.013°. The ZnO samples were provided on a Si (100) substrate and measured in reflection mode (Bragg–Brentano geometry). These measurements were performed for samples with ZnO SL thicknesses of ~10 nm and ~20 nm, as well as the resulting ZnO NWs of ~650 nm (Ø650) and ~200 nm (Ø200) in diameter, respectively.

Using Raman spectroscopy (Renishaw inVia, Wotton-under-Edge, UK), the structural characteristics of the as-grown ZnO NWAs were also examined at room temperature. The excitation power of a visible (wavelength of 532 nm) laser of 3 mW was utilized to measure the ZnO NWAs’ Raman spectra via a grating of 1800 l/m. The characterization was performed for as-grown Ø200 ZnO NWAs and Ø650 ZnO NWAs, comparing the results with bulk ZnO (001) crystal substrates purchased from MSE supplies, Tucson, AR, USA. Measurements were performed for the Zn-face and O-face of the bulk reference samples of sizes 5 mm × 5 mm × 0.5 mm (see Supplementary Figure S6).

### 2.5. Fabrication of ZnO-Based PENG

In general, the piezoelectric performance of a single NW improves with increasing aspect ratio. More specifically, the output voltage and output current of ZnO NWs are dependent on their length and diameter, respectively [47]. The piezoelectric output power increases with density within a particular range and drops beyond a particular density. This could be because of nearby NWs fusing together to create a single nanorod with an unnecessarily wider diameter, thereby indirectly decreasing its aspect ratio. Furthermore, a properly organized vertical array of NWs improves piezoelectric output and is less prone to shattering under stress [27].

**Figure 2:**
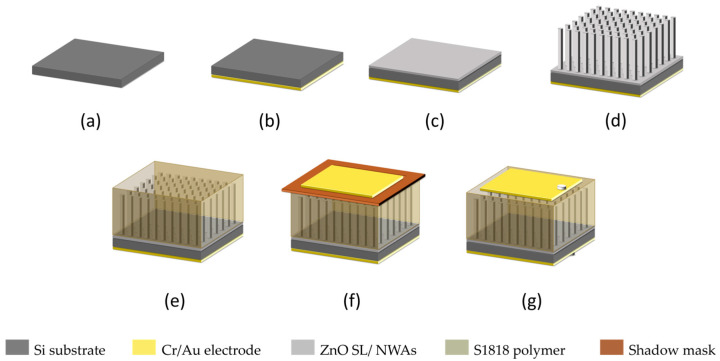
Fabrication steps of ultra-long ZnO NWAs on Si substrate for PENG: (**a**) Si substrate; (**b**) bottom Cr/Au contact electrode deposition; (**c**) Zn SL sputter coating and subsequent annealing; (**d**) TSG of a ZnO NWA; (**e**) NWs encapsulation in S1818 polymer matrix; (**f**) shadow mask and top Cr/Au contact electrode; (**g**) complete device with top and bottom Sn contact pads for measurement.

To study the influence of ZnO NW morphology on piezoelectric output, the ZnO NWA realized from our developed TSG system was used to fabricate a PENG. The fabrication steps are outlined in Figure 2.

In the fabrication process, *n*-type, P-doped Si wafers with resistivity of 1–5 Ω·cm and thickness of 525 ± 20 µm were utilized. The Si wafers were sliced into smaller pieces 20 mm by 15 mm in size to fit the heating element used in ZnO NW growth. A solution of 96% H_2_SO_4_ and 30% H_2_O_2_ with a volume ratio of 1:1 was used to clean the Si wafer, which was then dried with N_2_ gas. To avoid the creation of an oxide layer at the bottom of the silicon wafer, which would impede electrical conductivity, metal deposition was performed on the silicon wafer prior to substrate annealing. A 50 nm/500 nm thick Cr/Au to be used as bottom electrode was deposited on the back of the Si substrate by electron-beam (e-beam) evaporation (supplied by Leybold, Cologne, Germany). A S150B sputter coater (supplied by HHV Ltd., West Sussex, UK) was used for a 10 min DC sputtering of Zn metal on the Si surface, followed by a subsequent annealing in air at a temperature of 600 °C for 1 h, to produce the ZnO SL. CBD ZnO NW growth by the thermo-convective approach was performed using the TG1 container from our initial findings (see Supplementary Note S4). The samples were grown in a solution containing equal parts of zinc nitrate hexahydrate (Zn(NO_3_)_2_) and 0.025 M hexamethylenetetramine (HMT). The growth time was set for 24 h, at a growth temperature of 105 °C.

After the growth process, the ZnO NWs were embedded in S1818 polymer (supplied by Microresist Technology, Berlin, Germany) to enhance mechanical stability and avoid short-circuiting, thereby promoting the piezoelectric performance of the device [47]. To embed it in the polymer matrix, the sample was first dried with a stream of N_2_ gas after being cleaned with acetone. It was then placed on a heating plate at a temperature of 120 °C for 5 min while a stream of HMDS (hexamethyldisilane, purchased from Carl Roth GmbH + Co. KG, Karlsruhe, Germany) was applied as an adhesion promoter. S1818 polymer was then dropped on the NWAs and allowed to settle for 5 min, to ensure that the polymer penetrates the NWAs. Spin coating was then performed at a speed of 3000 rpm for 35 s. After that, the sample was put on a heating plate set at 90 °C for 90 s to remove the remaining solvent. A shadow mask was used to cover the sample’s surface, leaving a 15 mm by 15 mm region available for the top-electrode deposition that followed. This was necessary to avoid creating a short circuit at the sample’s edge. On top of the NWs embedded in S1818 polymer, a layer of Cr/Au (20 nm/200 nm) was deposited as top electrode by e-beam evaporation. After removing the mask, the top and bottom electrodes were soldered with Sn metal to serve as measurement contact points. To connect to the *I*-*V* analyzer (Semiconductor characterization system 4200 SCS, from Keithley), two Cu/Pb wires (Conrad Electronic SE, Berlin, Germany) were glued to the contact locations using conductive silver paste (purchased from Hanwuyou, Shenzhen, China) and extended from there to a printed circuit board (PCB). A mass of 91.5 g (about 0.9 N) was applied in dynamic compression mode to the nanogenerator device, and the piezoelectric output was measured with an SCS4200 *I*-*V* parameter analyzer (supplied by Keithley Instruments, Inc., Cleveland, OH, USA). For details of the measurement process and devices used, we refer to our earlier study [23].

## 3. Results and Discussion

### 3.1. Effect of SL Thickness on Morphology of ZnO NWAs

In the previous section, we discussed the general influences of SL thickness on the morphology of the resulting ZnO NWAs. We performed Zn sputtering on *n*-type Si for varied sputtering periods of 1, 5, 10, 15, 20, 30, and 40 min, followed by an annealing in air at 600 °C for 60 min. In this section, we analyze the relationship between the sputtering time and ZnO SL thickness. The ZnO SL thickness was measured using Laser Scanning Microscopy (3D Measuring Laser Microscope OLS5000, from Olympus Corporation, Tokyo, Japan) and atomic force microscopy (AFM, Dimension Icon) supplied by Bruker, Bremen, Germany. For this, step structures were prepared by selective-area removal of the SL layers using display foil as shadow masks. Considerable discrepancies were detected in the two sets of results, which could be associated with the different modes of optical and tactile probing (i.e., laser scanning and AFM). ZnO and Si have different reflectivity and absorbance, which may lead to a systematic error in the SL thickness values measured by LSM [48]. We therefore decided to use the values detected by AFM, which we consider to have provided more accurate results in this case. A description of the AFM measurements with results can be found under Supplementary Note S5.

**Figure 3:**
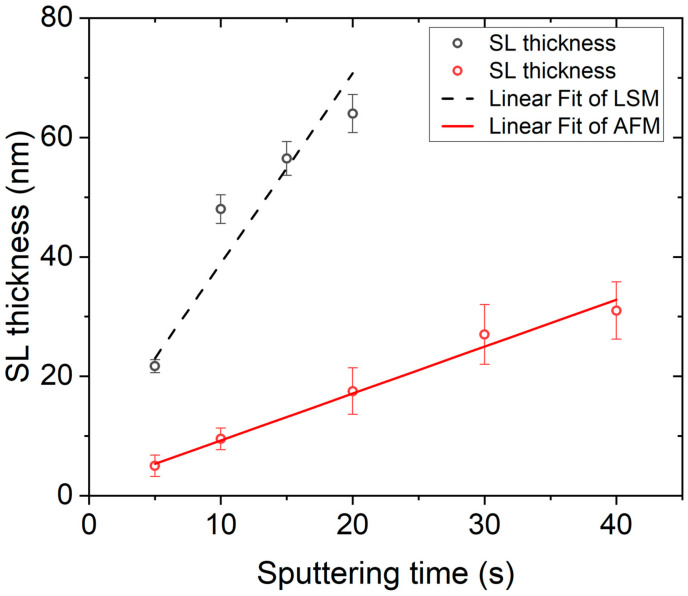
Experimental results of ZnO SL thickness showing a linear fit of both LSM (black) and AFM (red) measurements. Slope values of 3.2 ± 0.6 nm/ s for LSM and 0.78 ± 0.04 nm/ s for AFM were calculated from the linear fits, as well as 7.1 ± 5.5 nm and 1.4 ± 0.6 nm as *y*-intercept values for both LSM and AFM measurements, respectively.

The experimental results of SL thickness presented for analysis are for sputtering times of 5, 10, 15, 20, 30, and 40 min. For a sputtering time of 1 min, the SL was too thin and could not be detected either by LSM or AFM. Figure 3 shows a linear fit of results from both LSM and AFM measurements.

The primary mechanisms governing the SL growth are the Zn sputter-deposition process and the thermal oxidation of zinc in ambient air determined by the oxidation and transport of oxygen through the already grown ZnO on the Zn [49]. From the linear dependence of SL thickness on Zn sputtering time in Figure 3, we conclude that in the considered range of sputtering time, the deposited Zn was completely oxidized after annealing in air at 600 °C for 60 min.

**Figure 4:**
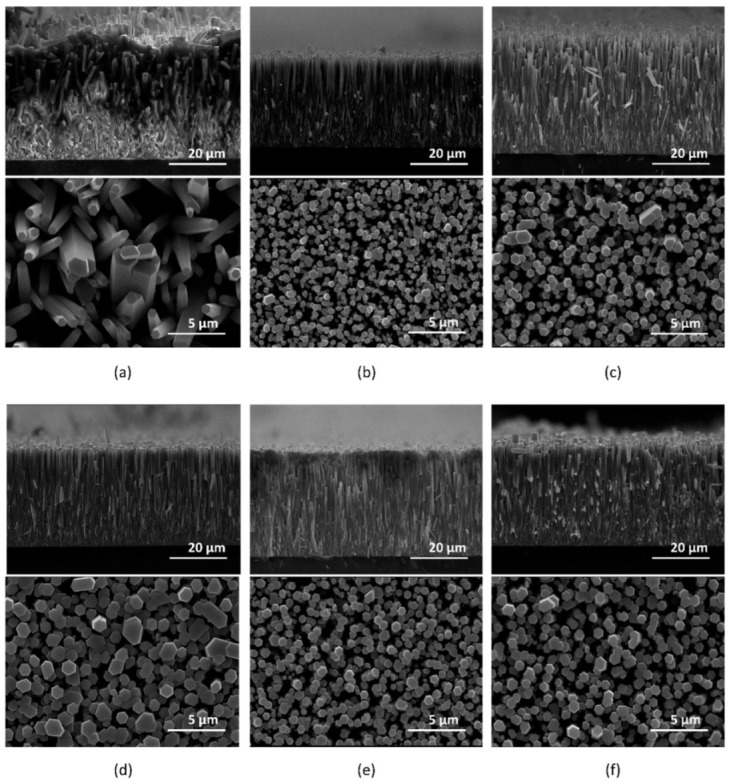
SEM micrographs of ZnO NWAs showing cross section and top views. The NWAs were synthesized by TSG, and they were of different ZnO SL thicknesses according to Zn sputtering times: (**a**) 1 min; (**b**) 5 min; (**c**) 10 min; (**d**) 20 min; (**e**) 30 min; and (**f**) 40 min.

On such SLs of different thickness on Si substrate, ZnO NWAs were grown based on the newly developed TSG method (Supplementary Note S1). Figure 4 displays inclined-view (upper rows, inclination angle = 90°) and top-view (lower rows) scanning electron microscopy (SEM) images of ZnO NWAs grown on ZnO SLs with varying thicknesses.

**Figure 5:**
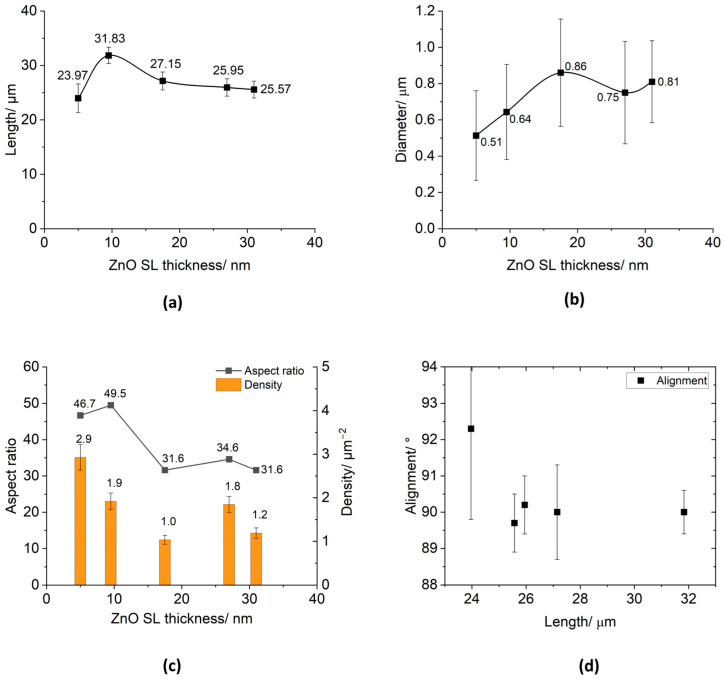
Morphology of ZnO NWAs by TSG for different SL thicknesses: (**a**) NW length vs. SL thickness; (**b**) NW diameter vs. SL thickness; (**c**) aspect ratio and density vs. SL thickness; (**d**) NW alignment vs. length.

As shown in the SEM images (cross section and top views) above, all the samples exhibit strong vertical alignment, except for Figure 4a, and b, showing SLs of Zn sputtered for 1 and 5 min, respectively. The diameter, length, and alignment of the NWs in the SEM images were measured using the image processing software ImageJ 1.53t. Table 1 presents the ZnO SL thickness values measured from 5 to 40 min sputtering times using AFM. Also presented in Table 1 are the quantitative details of the NW’s morphology, with corresponding graphs in Figure 5.

Table 1 shows that the ZnO NW length reached an astounding 35.3 ± 4.3 µm, although the calculated SL thickness was expected to be minimal (sputtering duration of 1 min). Not only could the NWAs for this sputtering time not be ordered vertically, but their length distribution over this cross section was highly uneven (±4.3 µm). Moreover, their erratic growth is evident from the cross-sectional view in Figure 4a. From the top view, it is evident that very large NWs with diameters greater than 1 µm emerged, while others appear smaller. This variation could be attributed to the NWs growing on an irregular or non-closed, island-like SL, thereby producing larger nanowire diameters during the experiment. In summary, due to the rather larger diameters of some of the NWs identified in the SEM micrograph, the density associated with the 1 min sputtering time could only reach (0.30 ± 0.03)/µm^2^. These observations suggest that the ZnO SL sample with a sputtering time of 1 min was unable to effectively produce the morphology of the NWs necessary for the optimal electrical performance of PENGs.

For an SL thickness of 5.0 ± 1.8 nm, Figure 5a shows a reduced NW length of ~24 µm. Its NW diameter also shows a reduction to 0.51 ± 0.25 µm. However, an increase to 2.9 ± 0.3 µm^−2^ in density is recorded, which demonstrates an acceptable length and diameter uniformity, as well as an orderly vertical alignment.

According to Table 1, the sample’s SL thickness reached 9.5 ± 1.8 nm after sputtering for 10 min. The density dropped to 1.9 ± 0.2 µm^−2^ as the diameter increased to ~0.64 µm. At this point, the NWA’s vertical alignment has seen significant improvement (Figure 5d), likewise its length, diameter, and growth uniformity.

When the sputtering time was extended from 20 to 40 min, the thickness of the ZnO SL increased from 9.5 ± 1.8 nm to 31.0 ± 4.8 nm. The NW length was reduced from 31.8 ± 1.5 µm to 25.6 ± 1.5 µm over this interval. Generally, the diameter of the NWs showed a tendency to increase as the thickness of the SL rose from 9.5 ± 1.8 nm to 31.0 ± 4.8 nm, although the homogeneity did not vary considerably. The error margins stated in Table 1 are taken from the average roughness parameter, *Rq* (Mean Square Roughness, RMS), calculated over an area of approximately 1.5 µm^2^ for each sample, following ISO 4287-1996 standards [50].

From the discussions so far, it is evident that highly homogeneous, vertically aligned ZnO NWAs can grow from an overly thin ZnO SL. The sample with the appreciably larger length and 90.0 ± 0.6° vertically aligned ZnO NWAs (31.8 ± 1.5 µm in length), largest aspect ratio of 49.5, and comparatively high density of 1.9 ± 0.2 µm^−2^, corresponded to an SL thickness of 9.5 ± 1.8 nm. Figure 5d depicts the various NW lengths and their respective vertical alignment angles in degrees (°).

Our result of a ZnO NW growth rate of 1.47 µm/h obtained with the thinnest SL (1 min sputtering time) is comparable to the ~2 µm/h reported by Chakraborty et al. [3], who employed seedless growth in a solution of equimolar concentrations of zinc nitrate hexahydrate and hexamethylenetetramine of 0.03 M at a temperature of 90 °C, which nearly corresponds to our settings. Aspect ratios of ~30 to ~50 (Table 1) are lower than those reported in their work. An important advantage of our synthesis method is the ability to pattern the substrate through lift-off for selective-area ZnO NW growth. A comparison of various growth methods reported in the literature is summarized in Table 2.

In the present study, we extended the investigation using XRD and Raman spectroscopy to obtain further insight into the crystal structure and the mechanical properties of long ZnO NWAs, as described in the following section.

### 3.2. Structural Properties of ZnO Seed Layer and ZnO NWAs

**Figure 6:**
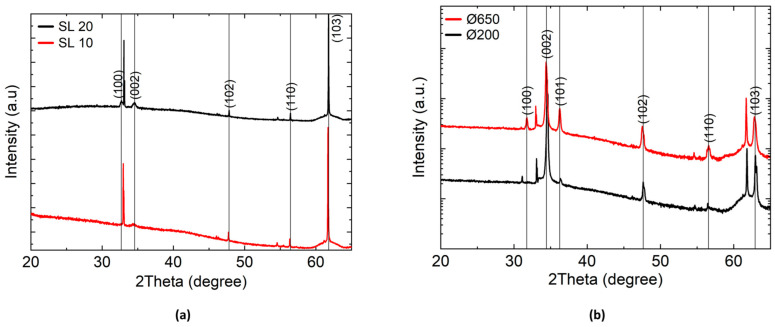
XRD diffractograms of (**a**) ZnO seed layers of 20 nm (SL 20) and 10 nm (SL 10) thickness; and (**b**) ZnO nanowires of 650 nm (Ø650) and 200 nm (Ø200) diameter, synthesized by thermo-convective solution-growth (TSG) and chemical bath deposition (CBD), respectively. As indicated by the colors of the XRD patterns, Ø650 and Ø200 correspond to SL 10 and SL 20, respectively.

The crystallinity and preferred growth direction of ZnO seed layers on the Si (100) substrate deposited by DC sputtering and subsequent annealing in air, as well as ZnO NWAs synthesized by CBD and TSG, were measured using XRD. The XRD pattern of ZnO seed layers with thicknesses of ~10 nm and ~20 nm (denoted as SL 10 and SL 20, respectively), as well as NW diameters of ~650 nm (Ø650) and ~200 nm (Ø200), corresponding to the pre-deposited SLs, are depicted in Figure 6. The Ø650 and the Ø200 NWA were grown on the SL 10 and the SL 20 seed layers, respectively; the growth parameters for the Ø200 NW and the Ø650 NW samples can be found in Supplementary Note S6. Each sample was measured under the same conditions.

The XRD (002) reflections for the ZnO SL in Figure 6a are located at 2*θ* = 34.49° and 2*θ* = 34.58° for SL thicknesses of 10 nm and 20 nm, respectively. They show lower intensities and broader shapes than the (102) and (110) peaks. In contrast, in Figure 5b the sharp reflections at 2*θ* = 34.39° for the Ø200 ZnO NWA and 2*θ* = 34.45° for the Ø650 ZnO NWA associated with the (002) lattice planes show that the ZnO nanowires have the highest elongation in this direction, indicating *c*-axis orientation of the NWs. This is in accordance with the alignment of the hexagonally shaped NWs perpendicular to the silicon surface visible in Figure 4 and Table 1, which were grown using TSG. According to previous studies, vertical alignment was also expected with our CBD ZnO NWAs [23,37]. In Figure 6b the relative intensity of the (002) reflection appears to be higher for the Ø200 NWA (by CBD) than for the Ø650 NWA (by TSG), consistent with findings that lower-diameter NWs exhibit better alignment [54,55,56]. In addition to the (002) reflection, other reflections were detected for both Ø200 nm and Ø650 nm samples (refer to Supplementary Note S6). The peak positions are basically in accordance with JCPDS standard (card no. 01-080-0074) for hexagonal (wurzite) ZnO. However, small shifts are detected, which can be assigned to compressive and tensile stresses in the NWs along lateral and vertical directions, respectively (see Supplementary Note S6).

The (002) direction shows the highest growth rate and surface energy compared to growth along other directions. According to the crystal growth orientation of ZnO films, the high-intensity (002) reflection relates to preferential *c*-axis growth of ZnO NWs perpendicular to the substrate surface, forming an aligned columnar structure [56]. Nevertheless, the non-vanishing reflections associated with the other lattice planes corroborate the impression from the SEM graphs (Figure 4) that the vertical alignment of the NWAs is not perfect. The significant intensities of the (103) reflections indicate preferred growth directions of NWs under specific low-inclination angles which may have been induced by the strong texture of the SL toward (103). Comparison of the observed XRD data with JCPDS data and various crystal parameters calculated [57,58] from the XRD patterns are presented in Supplementary Note S6.

**Figure 7:**
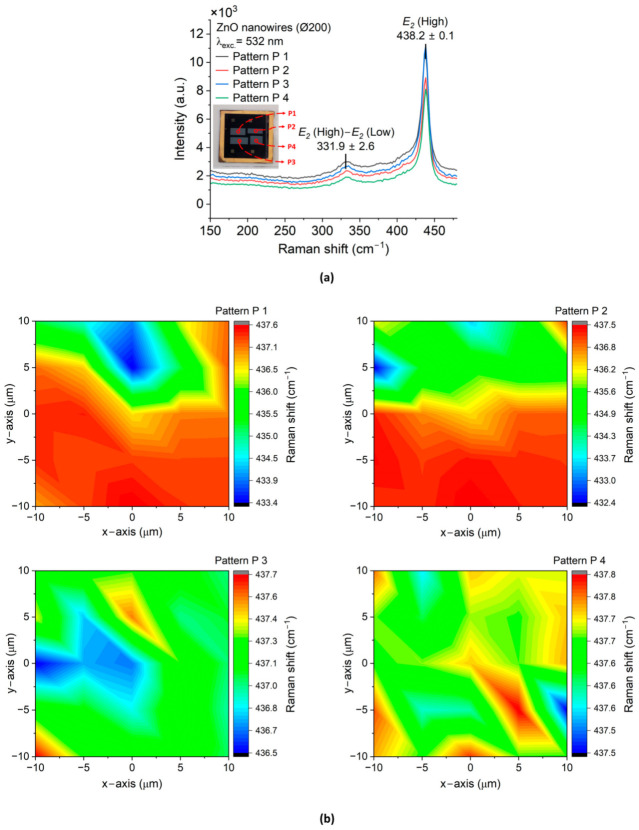
Raman spectroscopy of ZnO nanowires synthesized on (100) Si substrate by CBD: (**a**) Raman results of ZnO NWAs at the center of each patterned region, P1, P2, P3, and P4 (inset); (**b**) 2D mapping of the dominant Raman shift of *E*_2_ (high) for each patterned region after curve fitting. The Raman measurements were performed at room temperature, at a wavelength of 532 nm and with an excitation power of 3 mW.

**Figure 8:**
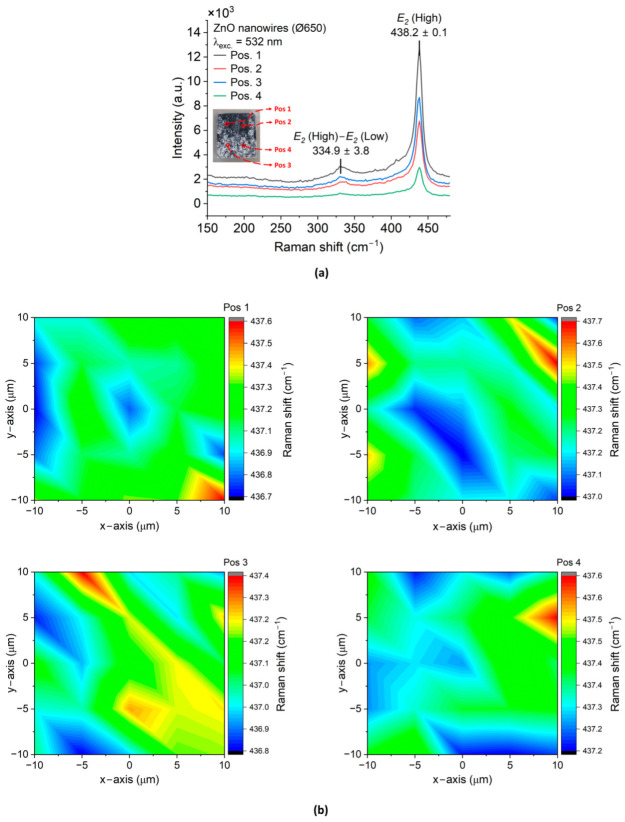
Raman spectroscopy of ZnO nanowires synthesized on (100) Si substrate by TSG: (**a**) Raman results of ZnO NWAs at each position on the sample (inset); (**b**) 2D mapping of the dominant Raman shift of *E*_2_ (high) for each position after curve fitting. The Raman measurements were performed at room temperature, at a wavelength of 532 nm and with an excitation power of 3 mW.

Figure 7 and Figure 8 depict the results of ZnO NWAs (Ø200 and Ø650, respectively) characterized by Raman spectroscopy.

As depicted in Figure 7a with the Ø200 ZnO NWA fabricated using standard CBD, a dominant peak was observed close to 438.2 ± 0.1 cm ^−1^ corresponding to the optical mode *E*_2_ (high, i.e., at high wave number) of ZnO (001). The same peak value was recorded for the Ø650 ZnO NWA, which was synthesized by the TSG method. The wide peaks at 331.9 ± 2.6 cm^−1^ (Figure 7a), and 334.9 ± 3.8 cm^−1^ (Figure 8a) could be ascribed to second order Raman scattering, which involves the acoustic phonon identified in CVD ZnO NWs [59]. It may alternatively be assigned to E2 (high)–E2 (low), which is related to the presence of hydrogen in interstitial sites or oxygen vacancies in ZnO [56]. From XRD it can be assumed that the NWs in both samples experienced some amount of strain, which is confirmed by comparing their peak wavenumbers in the scanned images to those for a reference bulk (001) ZnO crystal (refer to Supplementary Note S6). A variation lower than 0.9 cm^−1^ is visible in all patterns P1 to P4 of Ø200 while for Ø650 (Pos 1 to Pos 4), it is as large as 5.1 cm^−1^. The observed *E_2_* (high) peak’s linewidth for the Ø200 and Ø650 ZnO NWAs were around 10.5 cm^−1^ and 11.5 cm^−1^, respectively. These values are well comparable to the 9.2 cm^−1^ observed for the reference bulk (001) ZnO crystal, and to the 10.24 cm^−1^ and 9.32 cm^−1^ reported for ZnO NWAs by CBD on Si and ITO, respectively [56]. The 2D mapping of the dominant Raman peaks depicted in Figure 7 and Figure 8 indicate the dominant optical phonon mode of ZnO NWs with a distribution (peak shifts) within ±2.7 cm^−1^ for the Ø200 NWAs and ±0.5 cm^−1^, i.e., being more homogeneous, for the Ø650 NWAs. These findings indicate a slightly better crystallinity of the Ø200 ZnO NWAs, while strain and crystallinity of the Ø650 ZnO NWAs are more homogeneous across the measured arrays.

### 3.3. Output of PENG with Longer ZnO NWAs

**Figure 9:**
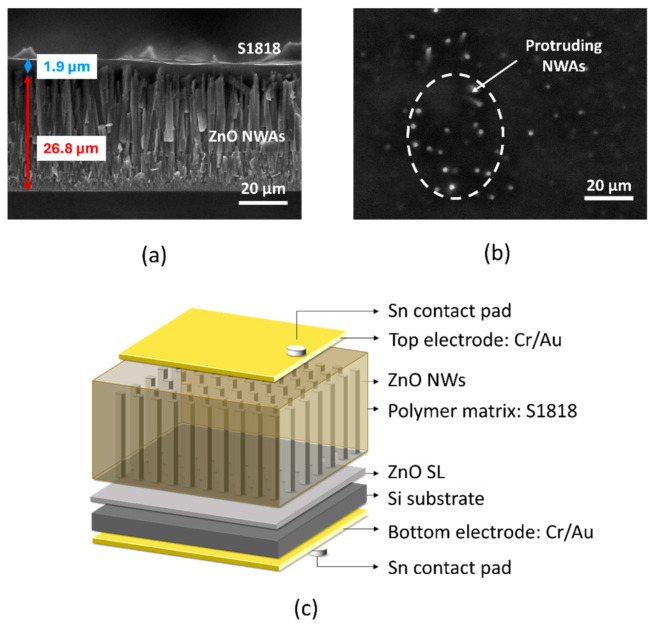
(**a**) SEM photograph of a densely grown ZnO NWA on (100) Si substrate, showing cross-sectional view; (**b**) top view of S1818 polymer layer spin coated on the NWA, showing protruding NWs; (**c**) schematic representation of a PENG device.

The cross section and top view of a ZnO NWA grown using TSG and embedded in polymer S1818, which was examined using an SEM, are displayed in Figure 9a and Figure 9b, respectively. Figure 9c depicts the schematic of a piezoelectric nanogenerator (PENG), which was fabricated from the NWA as described in Section 2.5 [23].

**Figure 10:**
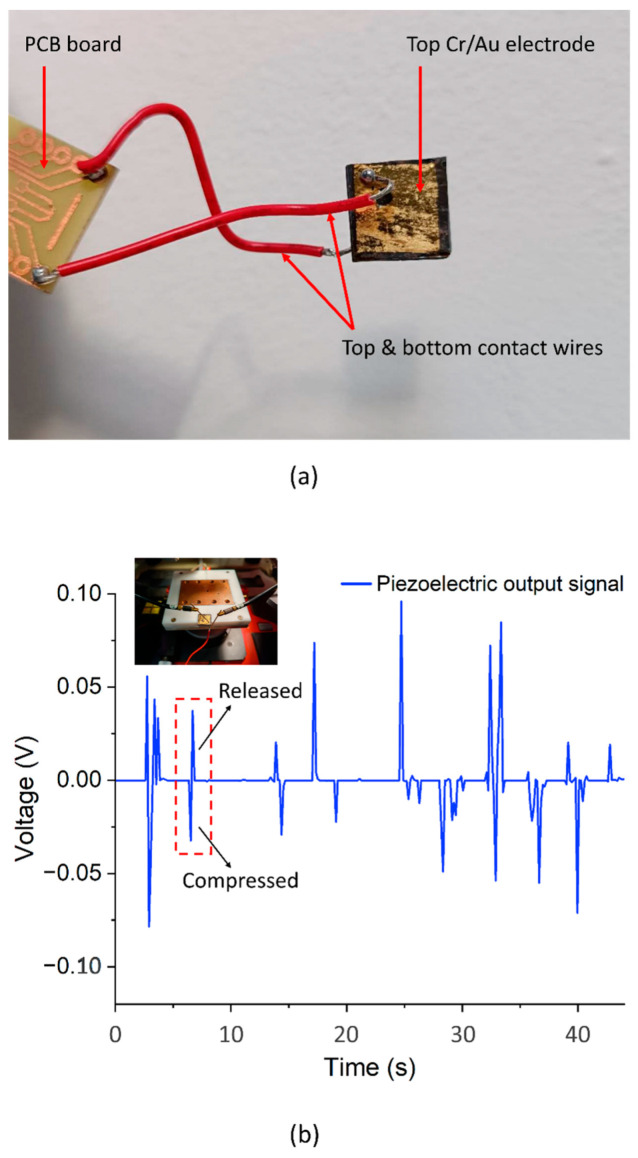
(**a**) Fabricated PENG device connected to a PCB board; (**b**) output voltage of PENG device measured with a Keithley SCS4200 *I*-*V* parameter analyzer. Inset picture: Device mounted on *I*-*V* analyzer stage for impulse-type compressive force application.

It is evident from the cross-sectional view in Figure 9a that the S1818 photoresist created a thin layer of around 2 µm thickness on top of the NWA rather than fully penetrating the space between NWs to the bottom. Furthermore, Figure 9b shows several protruding NWs piercing through the polymer layer surface. The top and bottom electrodes, which formed Schottky and Ohmic contacts, respectively [23], were connected to a Keithley SCS4200 *I*-*V* parameter analyzer. To excite a piezoelectric output of the device, a mass of 91.5 g, corresponding to a force of 0.9 N, was placed on/removed from the top of the device manually and repeatedly. A full description of the measurement process is detailed in our previous study [23]. The output voltage recorded from the device (Figure 10a) is presented in Figure 10b.

Figure 10b shows that the device can produce a typical piezoelectric signal when a vertical compressive force of roughly 0.9 N is applied to its surface through the mass piece. A negative potential is produced when the force is applied, and a positive potential is produced when the force is released. The non-uniform peaks detected in the output of the device can be attributed to the irregular manual loading and release of the applied mass (force). In that case, it can be expected that there was no uniform distribution of force on the nanowires’ tips. Also, not all the nanowires may have been in contact with the applied mass. An average peak output voltage of ~50.9 mV (calculated from absolute peak values) with a maximum peak value of ~95.9 mV was recorded. This was achieved with ZnO NW arrays with a length of ~26.8 µm, diameter of ~650 nm, and density of ~1.9 µm^−2^ grown on a (100) Si substrate with an area of 15 mm × 15 mm (Ø650 ZnO NWA). This output is substantially higher than the 11.2 ± 3.6 mV (maximum ~21.6 mV) reported for a ZnO NWA with a length of ~1.8 µm, diameter of ~200 nm, and density of ~20 µm^−2^, which was fabricated using CBD [23]. The difference in output voltage could be related to the longer NW dimensions with higher aspect ratio used in this current study [60].

**Figure 11:**
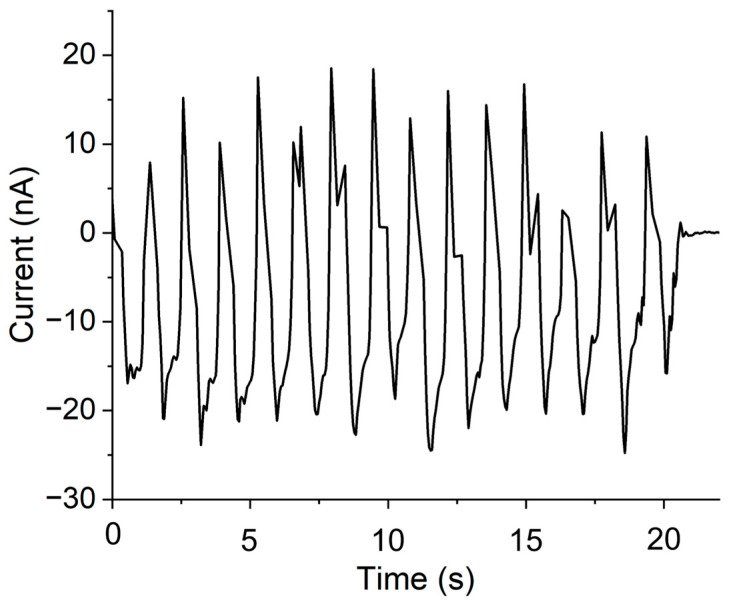
Current output measured with a Keithley SCS4200 *I*-*V* parameter analyzer at repeated application/release of 91.5 g mass/load.

To estimate the power output of the prefabricated PENG device, a resistive load of 100 kΩ (i.e., much lower than the input impedance of the Keithley analyzer of 10 TΩ) was connected across the circuit, and the short-circuit peak current, *I_sc-peak_*, was measured. To calculate the peak power, we used the equation

(1) $$P_{peak}=V_{oc-peak}\times I_{sc-peak}$$

where $V_{oc-peak}$ is the open-circuit peak voltage [23,61], measured to be 95.9 mV. From the measurement process with a repeated applied/released load of 91.5 g to the NWs’ top, we recorded a peak current output of 24.8 nA, as shown in Figure 11.

From the peak current of 24.8 nA and open-circuit peak voltage of 95.9 mV, we calculated a peak output power of 2.38 nW, which is much higher than the 0.017 nW reported in our earlier study [23]. The active volume, where the piezoelectric power was generated, can be considered as the area of the NWAs’ (15 mm × 15 mm) multiplied by the sum of the NWs’ height (~26.8 µm) and the thickness of the thin layer of polymer on top (~1.9 µm) [23,61]. From this, we calculated the power density per active volume to be 0.37 µW/cm^3^, which is nearly a factor of two higher than in our previous work with short NWs [23].

It should be noted that, in both our current and previous studies, the influence of the embedding polymer (S1818 for Ø650 and SU-8 for Ø200) forming a thin layer on top of the NWAs cannot be excluded. This influence is primarily due to the relative thickness of S1818 (1.5 µm to 2.5 µm) and high viscosity of SU-8 (45 cST at 25 °C). To overcome this challenge, it may be required to reduce their viscosities and to optimize the deposition processes (i.e., polymer settling time and spin speed). Work is in progress to elucidate this effect. A summary of results from similar works reported in the literature is presented in Table 3.

Due to the different experimental conditions in Table 3, the best piezoelectric performance of the various nanogenerators cannot be ascertained. Additionally, few studies have reported on ZnO NWs exceeding 10 µm in length. Hence, this study is intended to serve as a guide for the output performance improvement of such devices.

For robustness, long-term stability, and avoidance of potential short-circuiting, embedding NWAs in a polymer matrix offers advantages for improved output performance. Ou et. al. tested the long-term stability and reliability of their ZnO NW-based piezoelectric device by subjecting it to rigorous and continuous impacting cycles at a frequency 5 Hz for 30 h. After this period, the peak *V_out_* remained stable after an initial 10% drop [51]. At ambient pressure, ZnO may sublime at temperatures above 1000 °C [71]. In the case of NWs with carrier density greater than 10^18^ cm^−3^ at −196 °C, there is significant loss of piezo effect [72]. Bare ZnO NW operation has been investigated in humidities between 5% RH and 85% RH, with a voltage drop of about 33% observed at the higher humidity value. However, with surface modification, this drop is reduced to about 16% at 95% RH [73]. Therefore, embedding ZnO NWAs in a polymer matrix lessens the impacts of temperature and humidity on the device, while also improving its long-term performance stability and reliability.

## 4. Conclusions and Outlook

The standard chemical bath deposition (CBD) ZnO nanowire (NW) growth process was enhanced in this study to produce longer and vertically aligned ZnO NWAs based on the thermo-convective solution growth (TSG) method of CBD. The NWAs growth process was preceded by a Zn sputtering and subsequent annealing in air to form a ZnO seed layer (SL). Investigations demonstrate that the NW length obtained using the standard CBD reactor can reach 9.6 µm at a temperature of 105 °C and in a growth time of 24 h. With an improved setup using a thermo-glass container at the same temperature and growth time, the sample can achieve vertically aligned ZnO NWs with a length of ~26.8 µm. The growth rate in this case amounts to 1.12 µm/h, a 181% increase.

An SL of larger than ~5 nm in thickness was necessary for aligned NW growth in vertical orientation. The NW aspect ratio peaks at ~10 nm SL thickness (NW area density: 1.9 µm^−2^). The ZnO SL’s optimal thickness was found to be ~10 nm because it leads to high aspect ratio (49.5), NW area density (1.9 µm^−2^), vertical alignment of ZnO NWs (90.0 ± 0.6), and high growth rate (1.33 µm/h). This result is in line with the previously published research, which indicates a feasible range of 10 nm to 50 nm for the thickness of SLs by radio frequency sputtering of ZnO [45].

XRD and Raman spectroscopy characterizations reveal that the ZnO SLs and NWs grown by TSG exhibit high crystal quality, comparable to NWAs synthesized by CBD. XRD reflections with the highest intensities were observed for the (103) and (002) lattice planes for ZnO SLs and ZnO NWs, respectively, for NWAs grown by both TSG and CBD. Furthermore, dominant Raman peaks near 438.2 ± 0.1 cm^−1^, corresponding to the high optical mode *E*_2_ of ZnO (001), were the same for ZnO NWs synthesized by both standard CBD and TSG methods. Imaging of the Raman peak values of the patterned area of ZnO NWAs shows slightly lower values than bulk (001) ZnO, indicating local stress. The narrow linewidth observed confirms the high-quality crystallinity of the NWAs used in this investigation.

A sandwich structure, consisting of ZnO NW arrays on a Si substrate with a thin layer of S1818 polymer on top, and a top and bottom Cr/Au electrode, was used to fabricate a piezoelectric nanogenerator (PENG). The device, featuring ZnO NWs with a length of ~26.8 µm, a diameter of ~650 nm, and an active area of 15 mm × 15 mm on a (100) Si substrate, was fabricated using the TSG method. This device produced an average peak output voltage of ~50.9 mV, with a maximum of ~95.9 mV, power output of 2.38 nW, and a power density of 0.37 µW/cm^3^ under repeated compression and release of 0.9 N. These results indicate that long NWs with a high aspect ratio, fabricated by TSG, can provide much higher piezoelectric output voltage than standard NWs grown via CBD [23]. To further improve the output performance of our device, the ZnO NWAs can be area-selective grown through lithography patterning. By so doing, the patterned NWAs can be connected in series or in parallel for enhanced voltage and current outputs, respectively [23,68,74]. The fabrication process described in this work is simple and low-cost and can be scaled up to produce cheaper and reliable PENGs for energy harvesting and sensing applications.

In the next steps, different embedding polymers will be employed for the longer nanowire structures, and their effect on the PENG’s output will be compared. A newly built vibration stage will be used for measuring the piezoelectric output potential at varied vibration frequencies.

## Figures and Tables

**Table 1:** ZnO NW morphology according to sputtering times (SL thickness) and corresponding ZnO SL thicknesses. Ideal vertical alignment is defined as 90°.

Sputtering Time (min)	ZnO SL Thickness (nm)	Length-L (µm)	Diameter-Ø (µm)	NW Growth Rate (µm/h)	Aspect Ratio (L/Ø)	Density (µm^−2^)	Vertical Alignment (°)
1	-	35.3 ± 4.3	0.8 ± 0.4	1.5	42.0	0.3	92.4 ± 6.5
5	5.0 ± 1.8	24.0 ± 2.6	0.5 ± 0.2	1.0	46.7	2.9	92.3 ± 2.5
10	9.5 ± 1.8	31.8 ± 1.5	0.6 ± 0.3	1.3	49.5	1.9	90.0 ± 0.6
20	17.5 ± 3.9	27.2 ± 1.6	0.9 ± 0.3	1.1	31.6	1.0	90.0 ± 1.3
30	27.0 ± 5.0	25.9 ± 1.6	0.7 ± 0.3	1.1	34.6	1.8	90.2 ± 0.8
40	31.0 ± 4.8	25.6 ± 1.5	0.8 ± 0.2	1.1	31.6	1.2	89.7 ± 0.8

**Table 2:** Comparison of advantages and drawbacks of some ZnO NWA synthesis methods reported in the literature.

NW Growth Method	SL Deposition	NW Growth Rate (µm/h)	Max. NW Length (µm)	Advantages	Drawbacks	
Thermo-convective solution growth (target heating)	Dip coating	2.0	87.0	Low cost; low energy; low temperature; flexible substrates; fewer chemicals	Global heating; mass-transport limited growth; non-selective-area growth	[3]
Hydrothermal	Dip coating	2.4	12	Low cost; low temperature; fewer chemicals	More chemicals for ultra-long NWs; non-selective-area growth; mass-transport limited growth	[51]
Thermal evaporation	Seedless	-	-	Non-catalyst contamination	Capital intensive; high processing temperature; non-selective-area growth; non-flexible substrate	[1]
Physical vapor deposition	Pulse laser deposition	-	4	Non-catalyst contamination	Capital intensive, high processing temperature; non-selective-area growth; non-flexible substrate; non-homogenous growth	[52]
Chemical vapor deposition	RF sputtering	-	130	Non-catalyst contamination	High processing temperature; capital intensive; non-selective-area growth; non-flexible substrate	[8]
Hydrothermal	Spin-coating	0.8	2.4	Low temperature; low cost	Non-area-selective growth; mass-transport limited growth	[53]
Chemical bath deposition	DC sputtering	0.6	1.8	Low cost; low temperature; flexible substrate; selective-area growth	NWs growth in all areas; more chemicals for ultra-long NWs	[23]
Thermo-convective solution growth (target heating)	DC sputtering	1.5	35.3	Low cost; low energy; low temperature; flexible substrates; fewer chemicals; selective-area growth	Global heating	This study

**Table 3:** Power, voltage output, and applied force to ZnO NW-based piezoelectric nanogenerators.

Layers	NW L (µm)	NW D (nm)	Area (mm^2^)	Force	Load (*R*)	Peak V_out_	Peak Power	Power Density	
PC/ZnO/PC	12	250	30	impact, 75 Hz	1 MΩ	0.3 V	-	1.6 × 10^−3^ mW/cm^3^	[51]
Si/ZnO/SU-8	1.8	200	4 × 8	91.5 g (0.9 N) compressive	100 kΩ	21.6 mV	0.017 nW	0.22 µW/cm^3^	[23]
Si/ZnO/S1818	26.8	650	15 × 15	91.5 g (0.9 N) compressive	100 kΩ	95.9 mV	2.38 nW	0.37 µW/cm^3^	This study
Si/ZnO/PMMA	1.1	45	7	6 N @ 9 Hz compressive	5 MΩ	5.6 V	1.71 µW	38.47 mW/cm^3^	[62]
			7	6 N @ 500 Hz vibrative	5 MΩ	1.4 V	0.04 µW	0.9 mW/cm^3^	
Si/ZnO/PMMA	3	200	10	5 N compressive	1 MΩ	0.29 V	13 nW	85 µW/cm^3^	[63]
Si/ZnO/PDMS	1.5	200	20 × 20	0.451 N @ 10 Hz compressive	-	3 mV	-	-	[64]
p-Si/n-ZnO/PMMA	1.05	65	-	-	-	1.77 V	-	-	[65]
Si/ZnO/Au/PDMS	0.9	60	-	40 kHz	100 MΩ	2 V DC	4.59 pW	-	[66]
PDMS/Au/ZnO/Parylene C	0.59	70	10	3 N @ 5 Hz vibrative	100 MΩ	3.9 V	29 nW	-	[39]
PDMS/ITO/ZnO/Parylene C	0.70	70	10	3 N @ 5 Hz vibrative	100 MΩ	6.8 V	64 nW	-	
Phynox metal alloy/Au/ZnO/PMMA	3	170	30 × 6	fingertip impact	-	0.93 V	-	-	[12]
PET/Ni/ZnO/PMMA	2	200	25 × 25	10 Hz bending	1 MΩ	56 mV	-	-	[67]
Au/ZnO/PMMA	4	400	38	pressing; 0.5 Hz	-	15 mV	-	2.8 nW/cm^3^	[68]
Graphene/ZnO/PMMA	2.5	100	-	49 N compressive	-	0.17 V	-	0.004 µW/cm^3^	[69]
PAN/ZnO/PET	3	140	15 × 15	10 N, I Hz	0.45 MΩ	1.6 V	-	5.86 µW/cm^3^	[70]

## Data Availability

The data presented in this study are available on request from the corresponding author.

## Supplementary Materials

Supporting information associated with this article can be downloaded at: https://www.mdpi.com/article/10.3390/mi15101179/s1.
