# Supplementary material for: Thermo-Convective Solution Growth of Vertically Aligned Zinc Oxide Nanowire Arrays for Piezoelectric Energy Harvesting
Source: Micromachines (Basel). 2024 Sep 24;15(10):1179. doi: 10.3390/mi15101179 (PMC11509914; doi:10.3390/mi15101179)
Supplement: Supplementary file 1 [file micromachines-15-01179-s001.zip › micromachines-3160912-supplementary.pdf]

SUPPORTING INFORMATION

Thermo-convective Solution Growth of Vertically Aligned Zinc Oxide Nanowire Arrays for Piezoelectric Energy Harvesting

Frank Eric Boye Anang [1,2,*], Andam Deatama Refino [1], Gunilla Harm [1], Defang Li [1], Jiushuai Xu [1], Markys Cain [3], Uwe Brand [4], Zhi Li [4], Marion Görke [5], Georg Garnweitner [5,6], and Erwin Peiner [1,6]

1 Institute of Semiconductor Technology, Technische Universität Braunschweig, 38106 Braunschweig, Germany;
a.refino@tu-braunschweig.de; g.harm@tu-braunschweig.de; defang.li@tu-braunschweig.de; jiushuai.xu@tu-braunschweig.de; e.peiner@tu-braunschweig.de

2 Scientific Metrology Department, Ghana Standards Authority, P. O. Box MB245, Accra, Ghana

3 Electrosciences Ltd, Farnham, Surrey, GU9 9QT, United Kingdom;
markys.cain@electrosciences.co.uk

4 Surface Metrology Department, Physikalisch-Technische Bundesanstalt (PTB), 38116 Braunschweig, Germany; u.brand@ptb.de; zhi.li@ptb.de

5 Institute for Particle Technology, Technische Universität Braunschweig, 38104 Braunschweig, Germany; m.goerke@tu-braunschweig.de; g.garnweitner@tu-braunschweig.de

6 Laboratory for Emerging Nanometrology (LENA), Technische Universität Braunschweig, 38106 Braunschweig, Germany

* Correspondence: f.anang@tu-braunschweig.de; Tel: +49 151 4690 3701

Supplementary Note S1: Hydrothermal growth of ZnO nanowires (NWs)

Heterogeneous nucleation, or the depositing of aqueous ions onto a substrate, is the usual method used in chemical bath deposition (CBD) to create ZnO thin films. Hydrothermal growth techniques have the potential to dramatically lower processing costs [1] since in contrast to other approaches, they do not require high temperature fabrication or vacuum processing. For the fabrication of ZnO NWs, two agents are most frequently used: zinc nitrate ($Zn(NO_3)_2$), supplied by ThermoFisher, D-76870 Kandel, Germany and hexamethylenetetramine ($C_6H_{12}N_4$, HMT), purchased from SIGMA-ALDRICH CHEMIE GmbH, D-89555 Steinheim, Germany. The following are the reaction equations in aqueous solution, e. g., forming NH_3 and OH^- from HMT:

$$C_6H_{12}N_4 + 6H_2O \rightarrow 6HCHO + 4NH_3 \quad (S1.1)$$

$$NH_3 + H_2O \rightarrow NH_4^+ + OH^- \quad (S1.2)$$

$Zn(NO_3)_2$ provides Zn^{2+} ions and their reactions with NH_3 and OH^- in aqueous solution are given by:

$$Zn(NO_3)_2 \rightarrow Zn^{2+} + 2NO_3^- \quad (S1.3)$$

$$Zn^{2+} + 4OH^- \rightarrow Zn(OH)_4^{2-} \quad (S1.4)$$

$$Zn^{2+} + 4NH_3 \rightarrow Zn(NH_3)_4^{2+} \quad (S1.5)$$

From the zinc tetramine and tetrahydroxozincate ions are generation reactions of ZnO NWs, then synthesized according to:

$$Zn(NH_3)_4^{2+} + 2OH^- \rightarrow ZnO + 4NH_3 + H_2O \quad (S1.6)$$

$$Zn(OH)_4^{2-} \rightarrow ZnO(cluster) + H_2O + 2OH^- \quad (S1.7)$$

Before ZnO NWs can grow, the solution must become supersaturated. Once this happens, the molecular nucleation clusters that were formed initially break down quickly and the particles combine to form a thin film. This allows ZnO to grow on the surface of the sample through a heterogeneous reaction in the solution. By adhering to the non-polar sidewalls of the developing ZnO NWs and blocking Zn^{2+} from entering here, HMT can regulate the growth direction in the interim, leaving only the polar <0001> side available for growth. HMT primarily functions as a pH buffer by slowly releasing OH^- ions through thermal degradation, which aids in the formation of ZnO nanostructures during the hydrothermal process [2].

This hydrothermal approach is based on the chemical reactions described above to generate ZnO NWs. The standard hydrothermal synthesis procedure includes heating the entire chemical-bath system for an extended amount of time while the reactor vessel is inside an oven or on a heating plate, to synthesize the NWs. This approach allows for excellent temperature control during growth, but because the reactor's entire solution is heated, spurious/parasitic ZnO NWs are produced in regions of the substrate that are not required, thereby wasting precursor solution. In addition, there is a total waste of energy.

A promising technique for the controlled synthesis of vertically aligned and ultra-long NWs is the thermo-convective solution growth (TSG) based on targeted heating [3]. It is one of several research areas to enhance the hydrothermal synthesis process. In this technique, the ZnO NWs are formed on the substrate surface by localized heating caused by heat transfer from the heating element surface. The basic structure of the process involves fixing the substrate onto the surface of a heating element submerged in the solution. The chemical reaction in this process can be kept in its normal state by continuously supplying fresh reactants to the substrate surface. As a result, the precursor solution and energy loss are reduced by thermal convection. Figure S1 shows a setup of TSG for ZnO NWs growth.

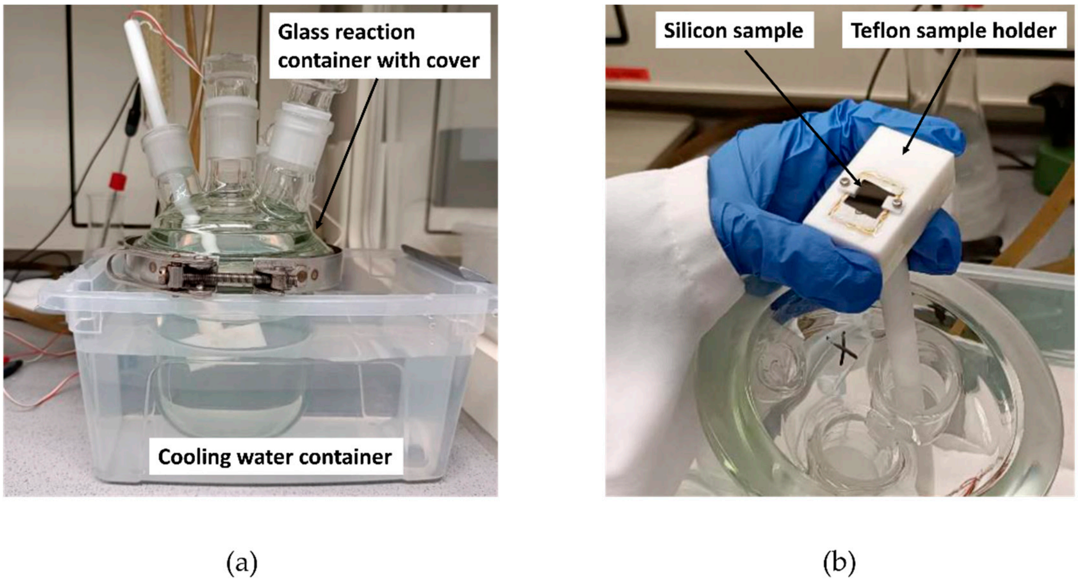

Figure S1: (a) Setup for thermo-convective solution growth (TSG) of ZnO NWs; (b) Teflon holder with sample attached to heating element.

Our group, Microelectromechanical Systems (MEMS), has constructed a homebuilt locally heated TSG system (Figure S1) for ZnO NWs using the above approach. Preliminary experiments on the growth of ZnO NWs using TSG showed ~30 μm length of ZnO NWs in 72 hours, which indicated a significantly lower vertical growth rate compared to the results of Abhisek Chakrabortey *et al*. [3]. However, it is our expectation that, by optimizing the growth temperature of the setup, larger aspect ratios ZnO NWs of vertical alignment and the growth of longer NWs based on published TSG method can be achieved. Furthermore, with our approach to ZnO SL deposition preceded by Zn sputtering and subsequent annealing in air, more regular shaped (not tapered), well aligned NWs will characterize their piezoelectric response.

Supplementary Note S2. Heating efficiency optimization

Measuring the temperature of the reaction solution in contact with the sample is necessary to accomplish the goal outlined in the preceding section. In our previous approach to ZnO NWs by TSG method, it

was not possible to measure the temperature from the heating element directly under perfect conditions because it is submerged in water in a closed environment during the experiment. As a result, the ideal scenario is approached using a new setup depicted in Figure S2.

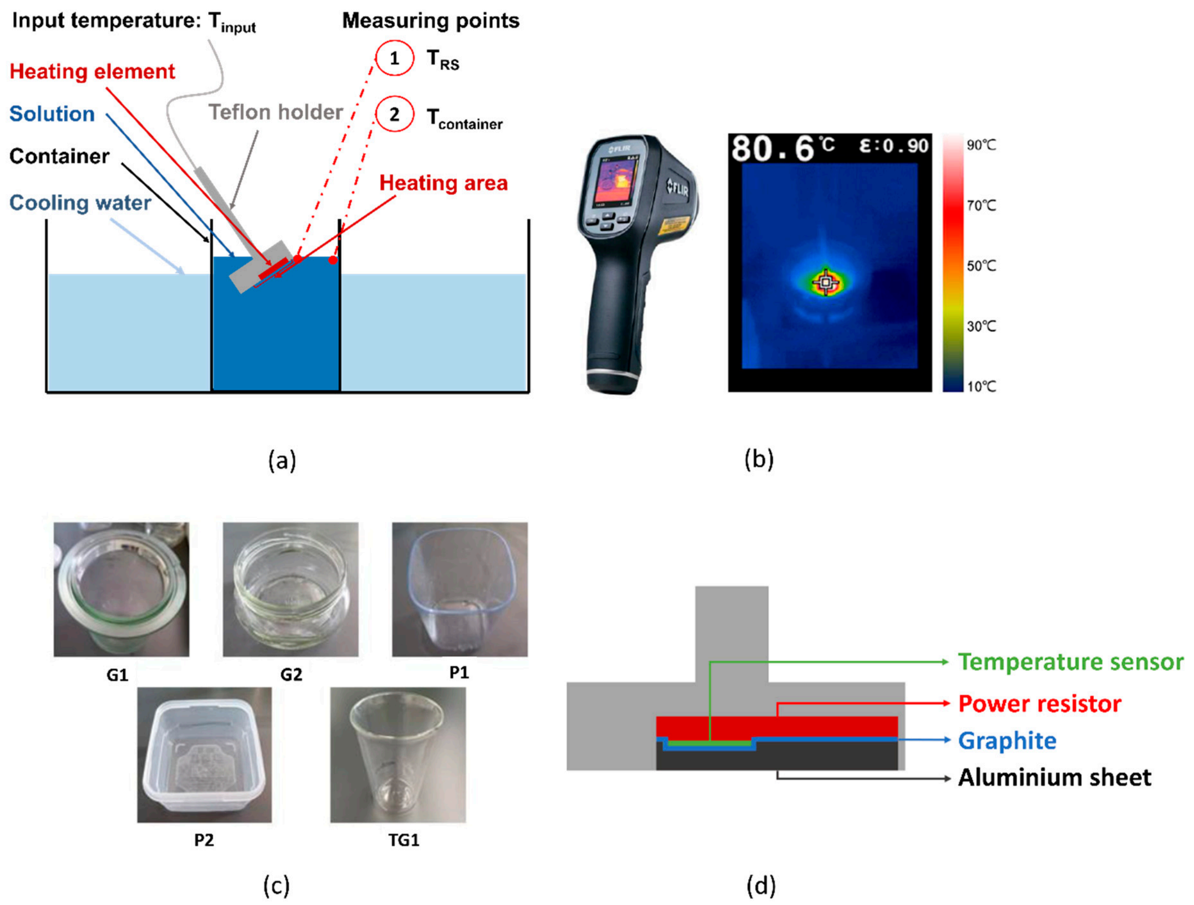

Figure S2: (a) Schematic of setup for optimizing local temperature conditions; (b) TG 165 infrared camera and image of measured temperature distribution around the heater in DI water in a reaction container; (c) containers used in the experiment; (d) Schematic of the heating system.

From Figure S2 (a), deionized (DI) water is initially used as the solution for temperature optimization. A Teflon holder is positioned at a 30° angle relative to the solution surface. To facilitate temperature monitoring, the heating element is submerged in the DI water and positioned as near to the water's surface as feasible. A power resistor (TEH 140, 140W, heating area: 22 mm × 16 mm, manufacturer: OHMITE) served as the heating element, and the temperature of the growth solution was measured using a thermocouple (TC) sensor that was fitted closely to the heating element. Two wires run from the top of the Teflon holder to the power resistor and the TC. One provides the heating current to the resistor and the other transmits the temperature signal. To improve heat dissipation, a sheet of graphite was placed over the resistor and the surface of the temperature sensor. A tiny temperature controller (not shown here) purchased from ENDA ET SERIES PID, SURAN Industrie Elektronik, terminates the

two wires which are used to set the heating temperature, as well as maintain it at a predetermined value. Two observation locations, namely the temperature near the heating element (temperature at reaction surface, T_{RS}) and the temperature close to the inner border of the container (temperature in container, $T_{\mathrm{container}}$), were measured using an infrared camera, TG 165, FLIR Corporation (Figure S2 (b)). By changing the containers (Figure S2 (c)) and raising the input temperature T_{input}, the measuring point T_{RS} indicates whether the central heating area has reached the ideal growing temperature for ZnO NWs. The temperature diffusion in the container's non-heated section can be seen at measuring point $T_{\mathrm{container}}$. This approach is aimed at preventing the consumption of too many chemicals resulting in a depletion of chemical precursors, by targeting ZnO NWs growth at the heated area, T_{RS}. For protection against corrosion, a 2-mm-thick aluminum sheet with the same dimension as the power resistor, is placed over the graphite layer. An adhesive epoxy was then used to fill the spaces created at the bottom of the Teflon holder. A schematic of the entire assembly which serves as the heating element is shown in Figure S2 (d) above. Table S1 presents the parameters of different containers used for the temperature measurement and heating efficiency optimizations in the TSG of ZnO NWs.

Table S1: Containers used in the investigation for temperature measurement and heating efficiency optimization.

Container ID	**G1**		**G2**		**P1**		**P2**		**TG1**	
Volume (ml)	500		250		500		250		250	
Material	Glass		Glass		Plastic		Plastic		Thermal glass	
Water cooling	Yes	No	Yes	No	Yes	No	Yes	No	Yes	No

Sealable containers of various sizes and materials were utilized as the means of investigating the effects of various containers on observation points T_{RS} and $T_{\mathrm{container}}$ used in the experiments. Each container was categorized into “with water cooling” (Yes) and “no water cooling” (No), with the goal of investigating the effects of water-cooling conditions on the temperature inside the container. At the start of the experiment, the input temperature, T_{input}, was set at 85°C. The infrared camera was then used to determine the temperatures at observation points T_{RS} and $T_{\mathrm{container}}$ once the input temperature stabilized. T_{input} was increased in steps of 5 °C until 105 °C, and for each step, the temperature data was captured using the infrared camera. The process was repeated for each container in accordance with the experimental procedure. To ensure optimal vertical growth of ZnO NWs, the following conditions are met.

Condition 1: Observation point $T_{\mathrm{RS}} \geq 90$ °C.

Condition 2: Observation point $T_{\mathrm{container}} \leq 40$ °C, as indicated in Figure S2 (a).

Besides the choice of a conducive container for NWs growth temperature optimization, it was necessary to investigate the heating efficiency of the proposed experimental setup based on the container of choice. This is aimed at determining the rate of temperature rise in the central heating area in the growth

container. In this investigation the heating rate in each container per time was observed. The maximum heating time was set at 150 minutes and the temperature at T_{input} was recorded every 5 minutes.

Supplementary Note S3. Results of temperature optimization

In the previous section, we have described the measurement process to optimize the heating process in our setup to have the most efficient temperature distribution on the substrate surface. For a low heat capacity of the solution, the container should be small and thus, the thermal conductivity through the container wall should be small as possible. By so doing, fast heating to the final $\mathrm{T}_{\text{input}}$ set temperature may be expected. This may also lead to elevated (but acceptable) container temperatures. The results from the various growth containers used in the investigation are presented in Figure S3.

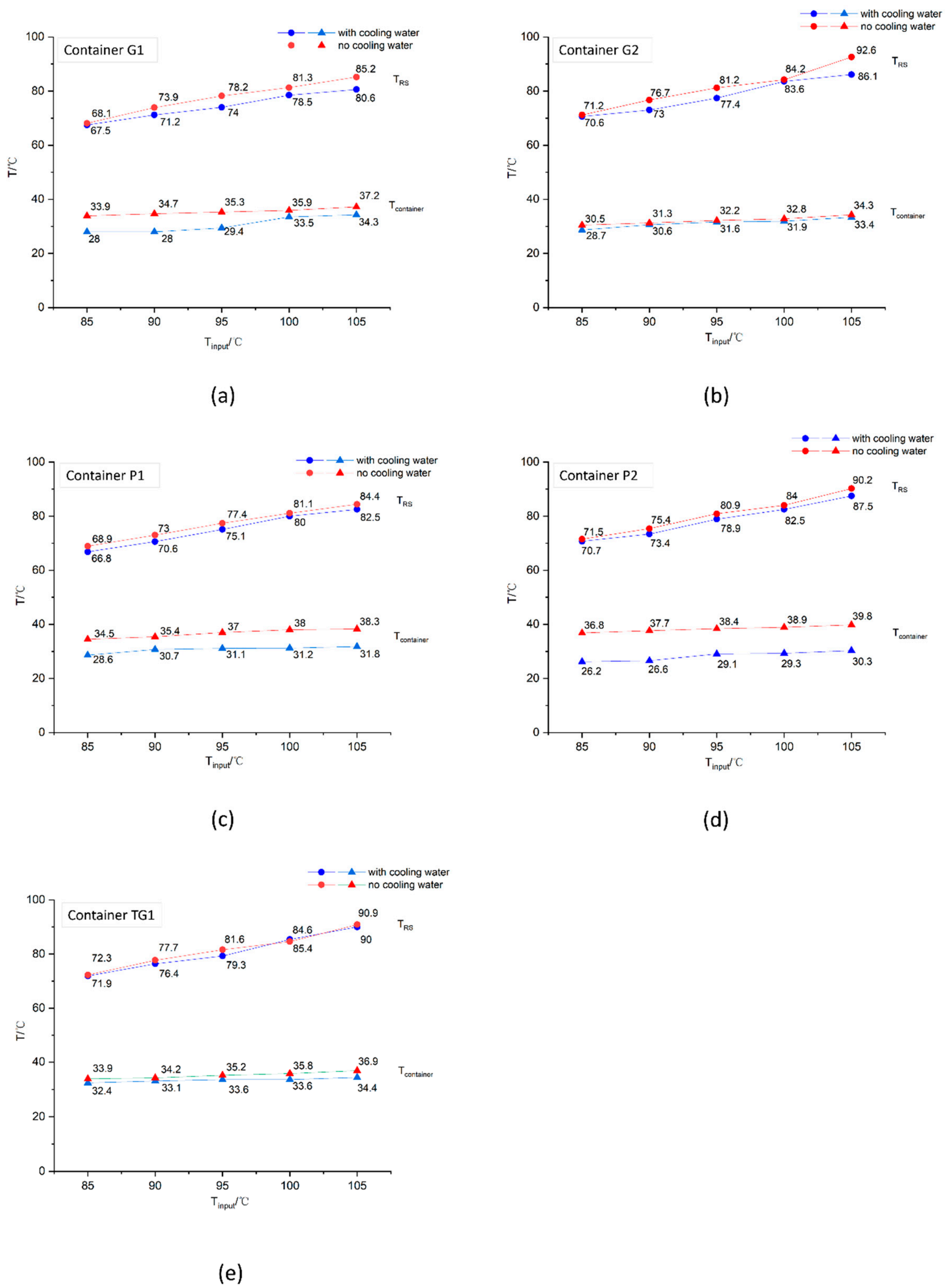

Figure S3. Results of reaction temperature with cooling water and without cooling water in the temperature optimization for different reaction containers of varied volumes: (a) Glass container G1 (500 ml); (b) Glass container G2 (250 ml); (c) Plastic container P1 (500 ml); (d) Plastic container P2 (250 ml); and (e) Thermal glass TG1 (250 ml).

The temperature control instrument's input temperature T_{input} and the interface's reaction temperature T_{RS} diverge significantly, as can be seen in Figure S3 (a). The reaction interface temperature in the G1 container without cooling water only reaches 85.2 °C even when the input temperature is at its maximum value of 105 °C. The same can be said with the other T_{input} setpoints with almost linear deviations ranging from (16.1 to 18.7) °C. With cooling water, it reaches 80.6°C with deviations from (16.5 to 21.5) °C, for the lower T_{input} setpoints. From the results presented, Condition 1 (TRS ≥ 90 °C) cannot be satisfied in any scenario. On the other hand, Condition 2 ($T_{container}$ < 40 °C) is satisfied since the measured temperature $T_{container}$ with cooling water and without cooling water all fall below 40 °C. However, for optimal and efficient heating of the substrate for NWs growth, both conditions must be met.

From Figure S3 (b), the reaction interface temperature in the G2 container without water cooling reaches 92.6 °C when T_{input} was set at 105 °C, fulfilling Condition 1. However, it only reaches 86.1 °C with water cooling. Whether or not the indirect heating area is water cooled, the temperature recorded at $T_{container}$ can satisfy condition 2. The disparity between the two curves (with cooling water and no cooling water) is less when compared to the G1 container. This demonstrates the impact of water-cooling when the container volumes differ. The cooling efficiency of the non-heated area is lessened when the solution volume is reduced. Comparing Figure S3 (a) and (b), it is evident that under the same cooling environment but with differing input temperatures, the G1 and G2 containers differ in terms of temperature. Regardless of the input temperature, the G2 temperature difference is less than the G1 temperature difference. This demonstrates that the temperature distribution within a solution in a glass container will be impacted by the volume of the solution, and that a container with a smaller capacity will improve the system's overall heating efficiency.

Utilizing container P1 (500ml), the measured data is displayed in Figure S3 (c) above. Only condition 2 is satisfied; condition 1 is not. The temperature difference (ΔT) of container P1 with water cooling is in the range of (18.2 – 22.5) °C, while without water cooling (16.1 – 20.6) °C. Comparing container G1 of the same volume of solution, the temperature range of container P1 is marginally narrower than that of the G1 container when water cooling is present. This suggests that the cooling water affects the glass material more in these circumstances and that the plastic containers are more advantageous for central heating efficiency. Without water cooling, the temperature deviations in both cases are similar.

While the temperature at observation point $T_{container}$ is always less than 40 °C satisfying conditions 2, Figure S3 (d) illustrates that observation point T_{RS} can only reach 90 °C without water cooling. We can see that there is not much of a change in the temperature measurement curves of observation point T_{RS} just like with G2 containers with the same solution volume laterally. However, the curves of their $T_{container}$ differ substantially. It is evident that the two curves nearly coincide in the temperature measurement experiment of G2 container because the non-direct heating region is not significantly

affected by the water-cooling condition. Nonetheless, there is a roughly 10 °C temperature difference between the two cases in the P2 container's non-heated section. This demonstrates that the plastic container's external cooling water has a greater influence on the temperature distribution in the unheated section, which helps to lower the solution consumption. The experimental conclusions show that, like glass containers, the temperature difference in P2 is smaller than in P1, regardless of the input temperature. This suggests that, in the case of plastic containers, the volume of the solution will influence the distribution of temperature in the solution, and that, to improve overall heating efficiency, containers with small capacities are advantageous. A comparison of the temperature differences shows that the data are not significantly different, indicating that the two materials have little effect on the temperature in the centralized heating area. Overall, the two materials, both in the case of small containers (250 ml) without cooling water, fulfil the condition of T_{RS} = 90 °C for T_{input} = 105 °C.

The 250 ml TG1 container is intended to investigate how the container structure affects heating efficiency. Its double layer glass construction provides the basis for its thermal insulation. The space between the two glass layers is cleared of air, creating a vacuum to prevent heat from escaping or entering the body thus creating a thermal insulation effect. The measurement results are displayed in Figures S3 (e). It can be observed that TG1, in contrast to the glass and plastic containers, can attain 90 °C at position T_{RS} when T_{input} is set at 105 °C, regardless of the water-cooling conditions. In other words, it meets condition 1. Additionally, it meets condition 2 (that is, $T_{container}$ < 40 °C) at the same time. The differences between the TG1 container and the glass and plastic containers are less and less pronounced. When compared to the other containers, the TG1 container exhibits excellent data performance and functions as a perfect experimental container that can be optimized to the system's ZnO NWs growth temperature. A summary of the comparison results is presented in Table S2.

Table S2. Summary of results for each container used in the investigation.

Container ID	**G1**		**G2**		**P1**		**P2**		**TG1**	
Volume (ml)	500		250		500		250		250	
Material	Glass		Glass		Plastic		Plastic		Thermal glass	
Water cooling	Yes	No	Yes	No	Yes	No	Yes	No	Yes	No
Condition 1	×	×	×	√	×	×	×	√	√	√
Condition 2	√	√	√	√	√	√	√	√	√	√

Condition 1: $T_{RS} \geq 90$ °C
Condition 2: $T_{container} \leq 40$ °C

It is evident from Table S2 that the containers G2, P2 can meet condition 1 (without cooling water). However, container TG1 meets both conditions, with and without cooling water. In summary, the system's overall heat dissipation, which includes indirect heating and central heating, are all influenced

by the external cooling water. Heat concentration in the central heating area is more suited for smaller capacity containers (250 ml).

Supplementary Note S4. Results of heating efficiency optimization

In another scenario, the relationship between heating time and input temperature (T_{input}) was also investigated for containers G2, P2 and TG1. The measured dependences were fitted using the two-phase exponential association equation (ExpAssoc)

$$y = y_0 + A_1\left(1 - \mathrm{e}^{-x/t1}\right) + A_2\left(1 - \mathrm{e}^{-x/t2}\right) \qquad \text{(S3.1)}$$

which describes the experimental trend very well, as depicted in Figure S4.

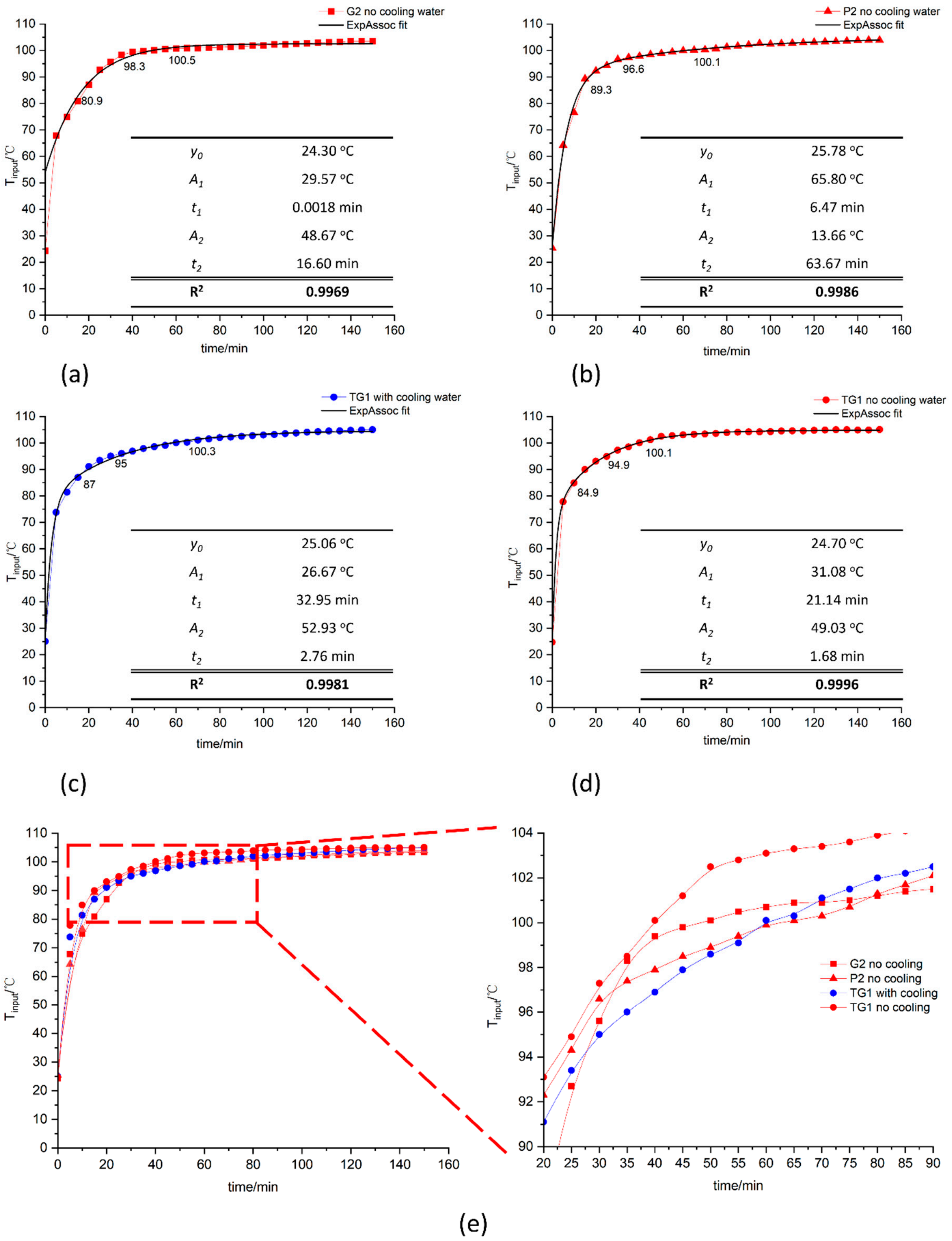

Figure S4. Measurement of T_{input} in relation to time and heating efficiency comparison: (a) temperature in container G2 with no cooling water; (b) temperature in container P2 with no cooling water; (c) temperature in container TG1 with cooling water; (d) temperature in container TG1 with no cooling water; (e) comparison of heating efficiency in containers G2, P2 and TG1 with magnified image.

The fitting curves' R^2 values in all four scenarios in Figure S4 are higher than 0.99, suggesting that they largely follow the rule of temperature rise. Though there are still some variations, the figure above shows that the heating efficiency under the four situations does not significantly change. One can distinguish three stages in the system heating curve. A quick ascent phase, starting from the start of heating and lasting for a few minutes (corresponding to the short-time component of the ExpAssoc fit), during which the T_{input} increases from room temperature to approximately 90 °C; a constant heating phase, lasting some tens of minutes (corresponding to the long-time component of the ExpAssoc fit), during which T_{input} is heated gradually between 90 °C and 100 °C; and finally, T_{input} value gradually approaches the limit value of 105 °C after more than 60 minutes.

Figure S4 (e) displays the combined temperature rise curves. It is evident that at the first stage, there is almost no variation in the rate of temperature rise for all four conditions. During the second stage, there is a gradual influence of the container's insulation performance on the input thermal efficiency. The fact that the TG1 container without water cooling may reach a higher temperature between 20 and 90 minutes is indicative of its ability to better keep the input temperature in the solution without losing heat, thanks to its two-layer glass structure. The system's heating efficiency remains competitive with the other three circumstances, even when subjected to water cooling. The system heating in all four cases has reached its limit in stage three, and the curves are beginning to overlap. As comparison nodes, four temperatures (90, 95, 100, and 105) °C are chosen to examine the rates at which the temperature is rising. The information is presented in Table S3.

Table S3. Time (in min) taken to reach node temperature and results of heating rate.

**Container ID**	**Temperature (°C)**				**Heating rate (°C/ min)**
	90	95	100	105	
	**Time (min)**				
G2 (no cooling water)	25	30	60	>150	1.67
P2 (no cooling water)	20	30	65	>150	1.54
TG1 (water cooling)	20	30	60	150	1.67
TG1 (no water cooling)	15	25	40	105	2.50

It is evident from the four scenarios that only in the TG1 container was the temperature inside the concentrated heating area ≥ 90 °C, with an input temperature that reached 105 °C in 150 minutes. Simultaneously, the four conditions can reach stage two in roughly an hour when looking at the whole heating time. When this point is reached, the heating efficiency is almost at its maximum and cannot be further increased, regardless of the technique employed. The system heating rates in each of the four scenarios, with 100 °C serving as the benchmark, are also shown in Table S3. It is evident that TG1's heating rate (2.5 °C/min) in the absence of water cooling is optimal. This is the situation with the TG1

container that has water cooling and the G2 container that does not, all having a heating rate of 1.67 °C/min. The P2 plastic container has the lowest heating rate of 1.54 °C/min.

The real growth period of ZnO NWs begins once the temperature reaches the predetermined level. However, the NWs are also slowly growing at low temperatures during the system's heating process. The input temperature needs to get to 105 °C to make the central heating area ≥ 90 °C. A lengthy heating time is necessary to maintain the input temperature steadily at this value. As a result, in the design of experiments, short growth durations of 3 hours should be avoided. An excessively high T_{input} >105 °C will result in an excessively long heating time and a significant growth time error. The case with water cooling should be chosen to minimize the formation of non-regional ZnO NWs and minimize the consumption of solution, given the necessity for long-term studies. Thus, the best option overall is to select TG1 container with water cooling.

Supplementary Note S5. Results of ZnO seed layer thickness

ZnO seed layer (SL) thicknesses, corresponding to Zn metal sputtering times of (1−40) min, were measured with an atomic force microscopy (AFM, Dimension Icon) supplied by Bruker, Germany. To measure the ZnO SL thickness and analyze the surface roughness, the AFM was used in a peak force controlling tap mode. To determine the average layer thickness, measurements were taken at 2−3 different positions along the sample. All measurements were performed at room temperature under ambient atmosphere. The average roughness parameter, Rq, was calculated for an area of ~1.5 μm^2 for each sample according to the standard ISO 4287/1-1997. The roughness values were taken as the measurement uncertainties associated with the SL thickness.

For comparison, SL thickness measurements were performed with a laser scanning microscopy (LSM, 3D Measuring Laser Microscope OLS5000, from Olympus Corporation, Japan) showing some discrepancy in the results, with the LSM results being considerably higher compared to those of AFM. This discrepancy could be associated with the different measurement modes of optical reflectance and tactile probing. We assume that LSM is affected by different reflectivity and absorbance of ZnO and Si, which lead to a systematic error of the SL thickness values measured using LSM. Nevertheless, both sets of measurement show linear dependences of SL thickness on sputtering time. The results of SL thickness measurements are presented in Figure S5 and Table S4 including those of AFM and LSM.

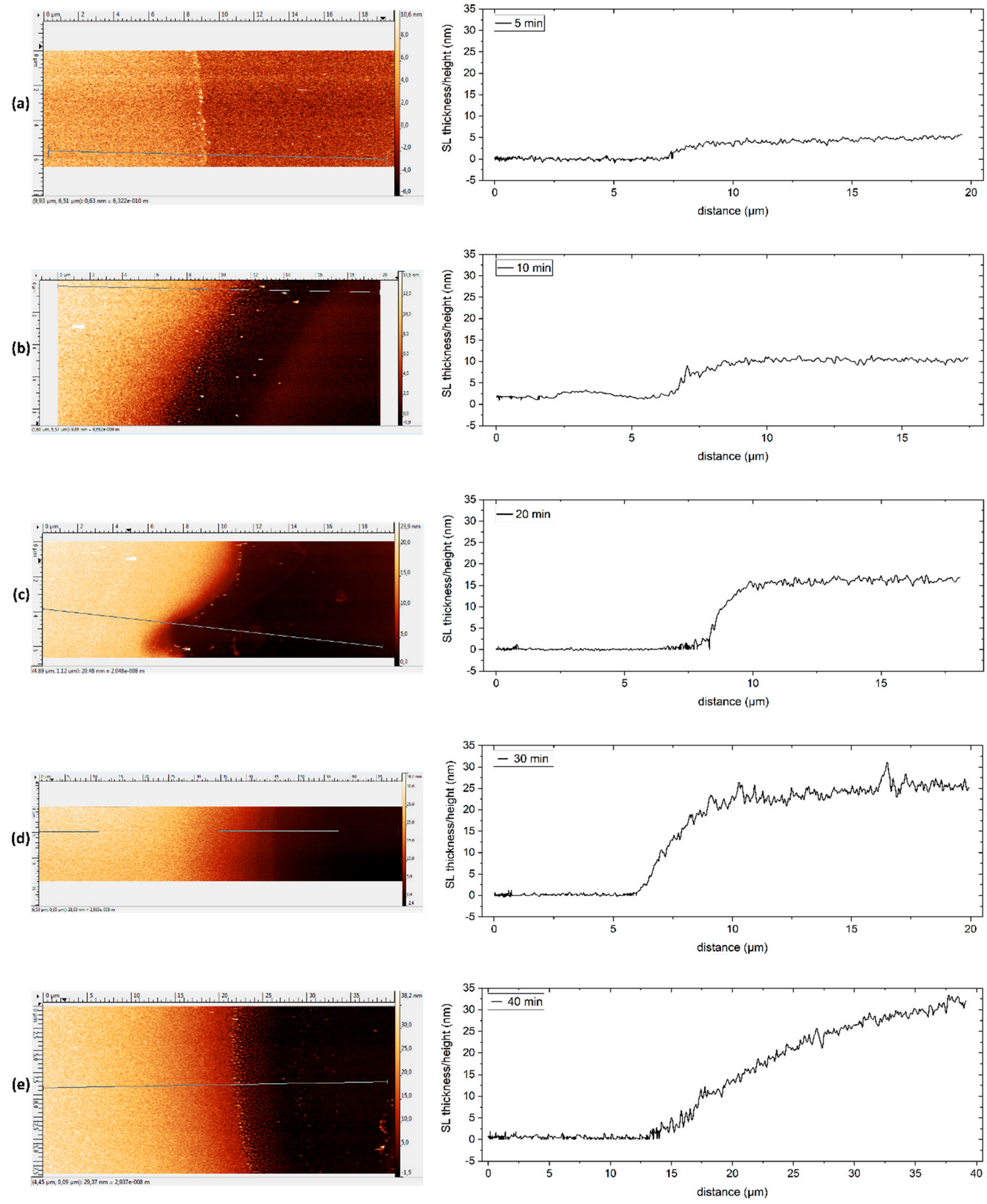

Figure S5. AFM scanned image of step height, and graph, indicating results of measured ZnO SL thicknesses for sputtering times of: (a) 5min; (b) 10 min; (c) 20 min; (d) 30 min; and (e) 40 min.

Table S4. Summary of results for AFM and LSM measurements of ZnO SL thicknesses for sputtering times of (5−40) min.

**Sputtering time (min)**	**ZnO SL thickness (nm)**		**Difference (%)**
	AFM	LSM	
**5**	5.0 ± 1.8	21.7 ± 4.6	76.9
**10**	9.5 ± 1.8	48 ± 6.9	80.2
**20**	17.5 ± 3.9	64.8 ± 8.0	73.0
**30**	27.0 ± 4.8	-	-
**40**	31.0 ± 0.5	-	-

Despite the discrepancy in both measurement methods by an average factor ~1.7, it can be observed that as the sputtering time increases, there is a corresponding increase in thickness of SL. The SL thickness for 1 min sputtering could not be measured in both cases since the thickness/step appeared to be too small to be detected.

Supplementary Note S6. Characterization of ZnO NWAs

The ZnO nanowires realised from both chemical bath deposition (CBD, Ø200 ZnO NWA) and thermo-convective solution-growth (TSG, Ø650 ZnO NWA) processes, on SLs of 100 nm and 10 nm thickness respectively, were characterised using *X*-ray diffraction (XRD) to determine the crystallinity and preferred growth orientations. Various parameters were measured, and their values compared to the standard JCPDS card no. 01-080-0074. The NWs length, diameter, density and vertical alignment are given by ~1.8 µm, ~200 nm, 5.9 μm^{-2} and 90.0 ± 0.9º, respectively for the Ø200 sample. Those for the Ø650 sample can be found in Table 1 of the main manuscript. All parameters were measured with ImageJ version 1.53t, and the densities determined from the SEM images using the software CountThings from photos, version 2.0.8949.

The lattice constants, *a,* and *c* of the hexagonal lattice can be calculated from the interplanar spacings (*d*-spacing) denoted by d_{khl} for the different *hkl* planes using the relation:

$$\frac{1}{d_{hkl}^2} = \frac{4}{3}\left(\frac{h^2+hk+k^2}{a^2}\right) + \frac{l^2}{c^2} \qquad \text{(S5.1)}$$

Using Eq. (5.1), *a,* and *c* for ZnO, can be calculated using the following expressions, which are correspondingly valid for the (100) and (002) planes, respectively

$$a = \frac{\lambda}{\sqrt{3} sin\theta_{100}} \qquad \text{(S5.2)}$$

$$c = \frac{\lambda}{sin\theta_{002}} \qquad \text{(S5.3)}$$

where λ is the wavelength of the X-ray source and θ_{100} and θ_{002} are the diffraction angles of the (100) and (002) peaks, respectively [5].

Using Bragg's law, d_{khl} can be calculated from all measured reflections corresponding to their respective positions according to the expression,

$$2d_{hkl}\sin\theta = n\lambda \tag{S5.4}$$

where λ is the wavelength of the X-ray source and θ is the angle of diffraction determined for the detected reflection of a certain (*hkl*) plane. From a and c the volume of the hexagonal cell (V) was calculated using the equation [5],

$$V = \frac{\sqrt{3}}{2}a^2c \tag{S5.5}$$

These values have been calculated and are presented in Tables S5 and S6. All the results presented are consistent with the standard JCPDS card no. 01-080-0074. We find almost perfect agreement of the SLs with the JCPDS card no. 01-080-0074, i. e., our SLs are free of strain, which was not expected regarding the different thermal expansion coefficients of ZnO and Si. Indeed, compressive stress was reported for thin ZnO films on silicon fabricated using the sol-gel method [6].

Table S5. Comparison of observed XRD results of ZnO NW arrays with the standard JCPDS data (no. 01-080-0074).

XRD reflection	2θ (degree)			Intensity (/%)			Interplanar spacing, d_{hkl} (nm)			Lattice constants (nm)			
	Observed		JCPDS	Observed		JCPDS	Observed		JCPDS	Observed		JCPDS	
hkl	Ø200	Ø650		Ø200	Ø650		Ø200	Ø650		*a*	*c*	*a*	*c*
1 0 0	31.724	31.794	31.732	0.29	3.45	56.4	0.2821	0.2814	0.2818	Ø200 = 0.3320<br>Ø650 = 0.3315	Ø200 = 0.5195<br>Ø650 = 0.5188	0.3257	0.5213
0 0 2	34.395	34.449	34.364	100	100	41.4	0.2607	0.2603	0.2607				
1 0 1	36.208	36.283	36.207	0.28	8.17	100.0	0.2481	0.2476	0.2479				
1 0 2	47.487	47.579	47.469	0.51	3.36	21.6	0.1915	0.1911	0.1914				
1 1 0	56.512	56.645	56.526	0.05	0.80	30.7	0.1628	0.1625	0.1627				
1 0 3	62.793	62.912	62.754	3.81	18.01	27.0	0.1480	0.1477	0.1479				

Table S6. Comparison of observed XRD results of ZnO SL with the standard JCPDS data (no. 01-080-0074). SL 10 and SL 20 denote the seed layers of ~ 10 nm and ~ 20 nm thickness, respectively (see Figure 6).

XRD reflection	2θ (degree)			Intensity (%)			Interplanar spacing, d_{hkl} (nm)			Lattice constants (nm)			
	Observed		JCPDS	Observed			Observed		JCPDS	Observed		JCPDS	
hkl	SL 10	SL 20		SL 10	SL 20	JCPDS	SL 10	SL 20		*a*	*c*	*a*	*c*
1 0 0	31.724	31.794	31.732	25.00	22.99	56.4	0.2821	0.2814	0.2818	SL 10 = 0.3257<br>SL 20 = 0.3250	SL 10 = 0.5213<br>SL 20 = 0.5213	0.3257	0.5213
0 0 2	34.409	34.409	34.364	25.34	21.45	41.4	0.2606	0.2606	0.2607				
1 0 2	47.498	47.548	47.469	18.69	14.36	21.6	0.1914	0.1912	0.1914				
1 1 0	56.511	56.645	56.526	13.55	11.64	30.7	0.1628	0.1625	0.1627				
1 0 3	62.813	62.854	62.754	100	100	27.0	0.1479	0.1478	0.1479				

Other parameters of the ZnO crystal structures were determined. The crystallite size (D) related to each diffractive peak can be calculated from Scherrer's equation,

$$D = \frac{K\lambda}{\beta\cos\theta} \qquad \text{(S5.6)}$$

and lateral strain (ε_{lat}) as well as vertical strain (ε_{vert}) according to [7]:

$$\varepsilon_{lat} = \frac{a - a_{JCPDS}}{a_{JCPDS}} \qquad \text{(S5.7a)}$$

$$\varepsilon_{vert} = \frac{c - c_{JCPDS}}{c_{JCPDS}} \qquad \text{(S5.7b)}$$

given β as the full width half maximum (FWHM) of the corresponding (*hkl*) reflection at the angle θ, and a constant shape factor $K = 0.89$. a_{JCPDS} and c_{JCPDS} are the reference lattice constants (JCPDS) [8], Gaussian fitting was used to determine the FWHM from the various reflections.

Vertical stress was calculated from strain using [6]:

$$\sigma_{vert} = -233 \times \varepsilon_{vert} \qquad \text{(S5.8)}$$

Table S7. Summary of other ZnO crystal parameters calculated from the XRD reflections of ZnO NWAs

XRD reflection	Crystallite size, D (nm)		Dislocation density $\times 10^{-3}$ (nm^{-2})		FWHM, β_{hkl} (degree)		Lattice volume $\times 10^{-1}$ (nm^3)		
							Observed		JCPDS
hkl	Ø200	Ø650	Ø200	Ø650	Ø200	Ø650	Ø200	Ø650	
1 0 0	77.293	102.583	0.1674	0.0950	0.09800	0.07957	0.040	0.040	0.048
0 0 2	50.990	60.719	0.3846	0.2712	0.14725	0.13562			
1 0 1	9.802	42.136	10.4074	0.5632	0.76238	0.19642			
1 0 2	27.155	39.840	1.3561	0.6300	0.26473	0.21577			
1 1 0	87.834	35.163	0.1296	0.8088	0.07888	0.25412			
1 0 3	90.204	129.303	0.1229	0.0598	0.07480	0.07087			

Table S8. Summary of other ZnO crystal parameters calculated from the XRD reflections of ZnO SLs

XRD reflection	Crystallite size, D (nm)		Dislocation density $\times 10^{-3}$ (nm^{-2})		FWHM, β_{hkl} (degree)		Lattice volume ($\times 10^{-1}$ nm^3)		
							Observed		JCPDS
hkl	SL 10	SL 20	SL 10	SL 20	SL 10	SL 20	SL 10	SL 20	
1 0 0	28.128	20.133	1.2639	2.4671	0.26889	0.40607	0.041	0.041	0.048
0 0 2	16.134	14.976	3.8416	4.4584	0.46553	0.54959			
1 0 2	82.932	120.433	0.1454	0.0689	0.08678	0.07134			
1 1 0	81.365	142.773	0.1510	0.0490	0.08512	0.06256			
1 0 3	70.654	134.930	0.2003	0.0549	0.09498	0.06829			

Regarding the measurement uncertainties of both our measurements and the JPCDS, the strain in the SLs calculated using the measured lattice constants and Equations (5.7a and b) is negligible. For comparison, with ZnO sol-gel thin-films compressive stress ranging from -0.58 GPa to -2.09 GPa was detected [6].

Both ZnO NW arrays Ø200 and Ø650 show larger values of a and smaller values of c than the JCPDS card no. 01-080-0074. Obviously, the NWs are under lateral compressive strain of 1.94 % to 1.77 % and vertical tensile strain of -0.35 to -0.48 %, for Ø200 and Ø650, respectively. For comparison lateral strain from -7.496 to -7.613 % and vertical strain from -0.044 to -0.170 % were reported for ZnO nanorods of 14 to 202 nm diameter by radio frequency (RF) magnetron sputtering [7]. Using Equation (5.8) and the measured strain we calculate vertical stress yielding values of 0.816 GPa and 1.118 GPa, for Ø200 and Ø650, respectively. These values indicate tensile stress, larger than the range of compressive stress of -0.235 GPa to -0.317 GPa reported with ZnO NW arrays by CBD on sol-gel-deposited SLs [8]. We

attribute this difference to the larger diameter and length our NWs, which amounts to ~650 nm and ~26.8 µm in case of the TSG growth. Furthermore, the strain-free (103)-textured SLs by Zn sputtering and oxidation used in this study instead of compressively stressed sol-gel ZnO SLs may influence the different stress states in the ZnO NWs.

The dislocation density for the different crystallographic planes of the ZnO NWAs and ZnO SLs were also calculated from the crystallite size using equation (5.8) and presented in Tables S6 and S7.

$$\delta = \frac{1}{D^2} \quad \text{(S5.8)}$$

The dislocation density (δ) indicates the dislocation line lengths per unit volume in the crystal. The calculated values are very low indicating good quality crystalline material formation. The difference in dislocation density for each crystal plane is because of the crystal growth anisotropy [9]. In the (002) plane of the synthesized ZnO NWs (Ø200 and Ø650), the calculated crystallite size values at ~50 nm and ~60 nm correspond to dislocation densities of (0.3846×10^{-3} and 0.2712×10^{-3}) nm^{-2} respectively. These values appear to be lower than the 1.662×10^{-3} nm^{-2} reported for ZnO NWAs on (100) Si substrate [8], indicating lesser defects in our structures. However, for our ZnO SLs, the calculated dislocation densities in the (002) plane are much higher (3.8416×10^{-3} nm^{-2} and 4.4584×10^{-3} nm^{-2}), corresponding to crystallite sizes of ~16 nm and ~15 nm for SL 10 and SL 20 seed layers, respectively. Such large dislocation densities can be assigned to the mismatch of the lattice between (100) Si and ZnO. Nevertheless, dislocation densities are reduced by an order of magnitude in the NW grown on top of the SLs. The results presented are in good agreement with that presented in literature which indicates that the ZnO material used in this work have relatively high crystal quality.

The ZnO nanowires of different dimensions (Ø200 and Ø650 ZnO NWAs, ~1.8 µm/ 26.8 µm lengths) synthesized by CBD and TSG growth methods, respectively, were further characterized with Raman spectroscopy to determine their crystallinity and strain. To compare the results, Raman measurement was performed for a reference bulk ZnO (001) crystal substrate, the results of which are presented in Figure S6.

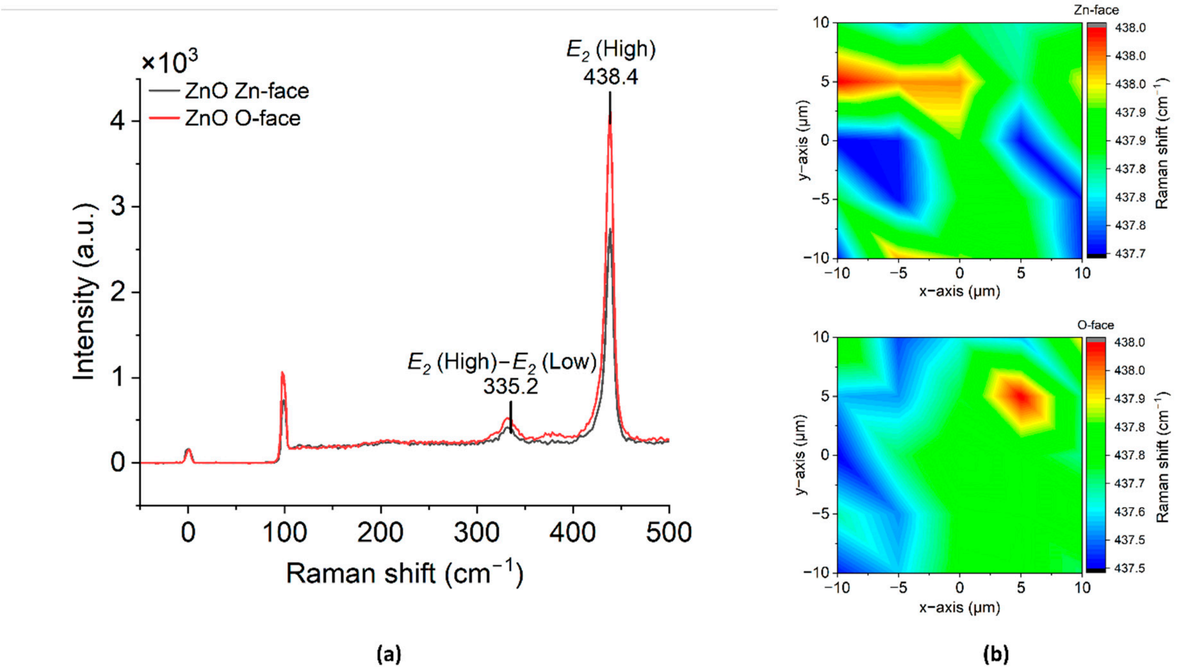

Figure S6. Raman spectroscopy of bulk (001) ZnO crystal substrate; (a) Raman results for bulk Zn-polar and O-polar (001) ZnO surfaces; (b) 2-D mapping (after curve fitting) of the dominant Raman shift of Zn-polar and O-polar (001) ZnO surface at 438.4 cm^{-1}. The Raman measurements were performed at room temperature, at a wavelength of 532 nm and with an excitation power of 3 mW.

Figure S6 (a) shows a dominant Raman peak at 438.4 cm^{-1} for the Zn-polar and O-polar (001) ZnO surface which determines the growth direction of the bulk ZnO. A 2-D mapping of the dominant Zn-polar and O-polar (001) ZnO Raman peak is depicted in Figure S6 (b) indicating the dominant optical phonon mode of unstrained bulk ZnO with a homogeneous distribution within ± 0.15 cm^{-1} for the Zn-polar and ± 0.25 cm^{-1} for the O-polar.

References

[1] Poulin-Vittrant, G.; Dahiya, SA. S.; Boubenia, S.; Nadaud, K.; Morini, F.; Justeau, C. "Challenges of low-temperature synthesized ZnO nanostructures and their integration into nano-systems," *Materials Science in Semiconductor Processing*, vol. 91, pp. 404–408, 2019, doi: 10.1016/j.mssp.2018.12.013.

[2] A. Sugunan, H. C. Warad, M. Boman, and J. Dutta, "Zinc oxide nanowires in chemical bath on seeded substrates: Role of hexamine," *J Sol-Gel Sci Technol*, vol. 39, no. 1, pp. 49–56, 2006, doi: 10.1007/s10971-006-6969-y.

[3] Chakraborty, A.; Orsini, A.; Kar, J.P.; Gatta, F.; Khan, U.; Falconi, C. Ultra-efficient thermo-convective solution-growth of vertically aligned ZnO nanowires. *Nano Energy* **2022**, *97*, 107167

[4] Panchal, V., Yang, Y., Cheng, G., Hu, J., Kruskopf, M., Liu, C-I., Rigosi, A. F., Melios, C., Walker, A. R. H., Newell, D. B., Kazakova, O. and Elmquist, R. E., "Confocal laser scanning microscopy for rapid optical characterization of graphene," *Communications physics*, vol. 1, 2018, doi: 10.1038/s42005-018-0084-6.

[5] A. Singh and H. L. Vishwakarma, "Study of structural, morphological, optical and electroluminescent properties of undoped ZnO nanorods grown by a simple chemical

precipitation," *Materials Science-Poland*, vol. 33, no. 4, pp. 751–759, 2015, doi: 10.1515/msp-2015-0112.

[6] Moussa, B. B.; Lajnef, M,; Jebari, N.; Villebasse, C.; Bayle, F.; *et al.,* "Synthesis of ZnO sol-gel thin-films CMOS-Compatible," *RSC advances*, vol. 11, no. 37, pp. 22723–22733, 2021, doi: 10.1039/d1ra02241e

[7] A. F. Abdulrahman, A. A. Barzinjy, S. M. Hamad, and M. A. Almessiere, "Impact of Radio Frequency Plasma Power on the Structure, Crystallinity, Dislocation Density, and the Energy Band Gap of ZnO Nanostructure," *ACS omega*, vol. 6, no. 47, pp. 31605–31614, 2021, doi: 10.1021/acsomega.1c04105.

[8] B. M. Nizar, M. Lajnef, J. Chaste, R. Chtourou, and E. Herth, "Highly C-oriented (002) plane ZnO nanowires synthesis," *RSC advances*, vol. 13, no. 22, pp. 15077–15085, 2023, doi: 10.1039/d3ra01511d.

[9] S. Sahu and P. K. Samanta, "Peak Profile Analysis of X-ray Diffraction Pattern of Zinc Oxide Nanostructure," *J. Nano- Electron. Phys.*, vol. 13, no. 5, 05001-1-05001-4, 2021, doi: 10.21272/jnep.13(5).05001.
